# Condensin I protects meiotic cohesin from WAPL-1 mediated removal

**DOI:** 10.1371/journal.pgen.1007382

**Published:** 2018-05-16

**Authors:** Margarita R. Hernandez, Michael B. Davis, Jianhao Jiang, Elizabeth A. Brouhard, Aaron F. Severson, Györgyi Csankovszki

**Affiliations:** 1 Department of Molecular, Cellular, and Developmental Biology, University of Michigan, Ann Arbor, MI, United States of America; 2 Center for Gene Regulation in Health and Disease and Department of Biological, Geological and Environmental Sciences, Cleveland State University, Cleveland, OH, United States of America; University of Iowa, UNITED STATES

## Abstract

Condensin complexes are key determinants of higher-order chromatin structure and are required for mitotic and meiotic chromosome compaction and segregation. We identified a new role for condensin in the maintenance of sister chromatid cohesion during *C*. *elegans* meiosis. Using conventional and stimulated emission depletion (STED) microscopy we show that levels of chromosomally-bound cohesin were significantly reduced in *dpy-28* mutants, which lack a subunit of condensin I. SYP-1, a component of the synaptonemal complex central region, was also diminished, but no decrease in the axial element protein HTP-3 was observed. Surprisingly, the two key meiotic cohesin complexes of *C*. *elegans* were both depleted from meiotic chromosomes following the loss of condensin I, and disrupting condensin I in cohesin mutants increased the frequency of detached sister chromatids. During mitosis and meiosis in many organisms, establishment of cohesion is antagonized by cohesin removal by Wapl, and we found that condensin I binds to *C*. *elegans* WAPL-1 and counteracts WAPL-1-dependent cohesin removal. Our data suggest that condensin I opposes WAPL-1 to promote stable binding of cohesin to meiotic chromosomes, thereby ensuring linkages between sister chromatids in early meiosis.

## Author summary

During the early stages of meiosis, duplicated copies of chromosomes must be held together, and homologous chromosomes must pair to ensure formation of sperm and oocytes with the correct number of chromosomes. A protein complex called cohesin is essential for this process. A related complex called condensin is responsible for the formation of condensed and rigid chromosomes. Our study identifies a novel role for condensin in cohesin protection. Cohesin complexes eventually need to be removed from chromosomes in later meiosis. In condensin mutants, a significant portion of cohesin is removed prematurely. Condensin protects cohesin from Wapl, a protein known to remove cohesin from chromosomes in early mitosis and meiosis. Human oocytes arrest in prophase I of meiosis and remain at this stage for several decades with little turnover of cohesin. Deterioration in cohesin function is thought to contribute to the decrease in oocyte quality with age. Our data suggest that condensin may counteract this decline and thereby promote reproductive health.

## Introduction

Meiosis is a specialized form of cell division in which one round of DNA replication is followed by two rounds of chromosome segregation to produce haploid gametes. Critical to this process is the timely establishment and sequential release of connections between homologous chromosomes and sister chromatids. During mitosis, cohesin complexes tether sister chromatids from S-phase until the complete release of sister chromatid cohesion (SCC) at anaphase onset. In contrast, stepwise release of meiotic SCC allows separation of homologs in meiosis I and sisters in meiosis II. In addition, meiotic cohesin is required for assembly of the synaptonemal complex (SC) between homologous chromosomes (synapsis) and for interhomolog crossover recombination. Underlying the unique functions of cohesin during gametogenesis, meiotic and mitotic cohesin complexes differ both in subunit composition and regulation.

**Fig 1:**
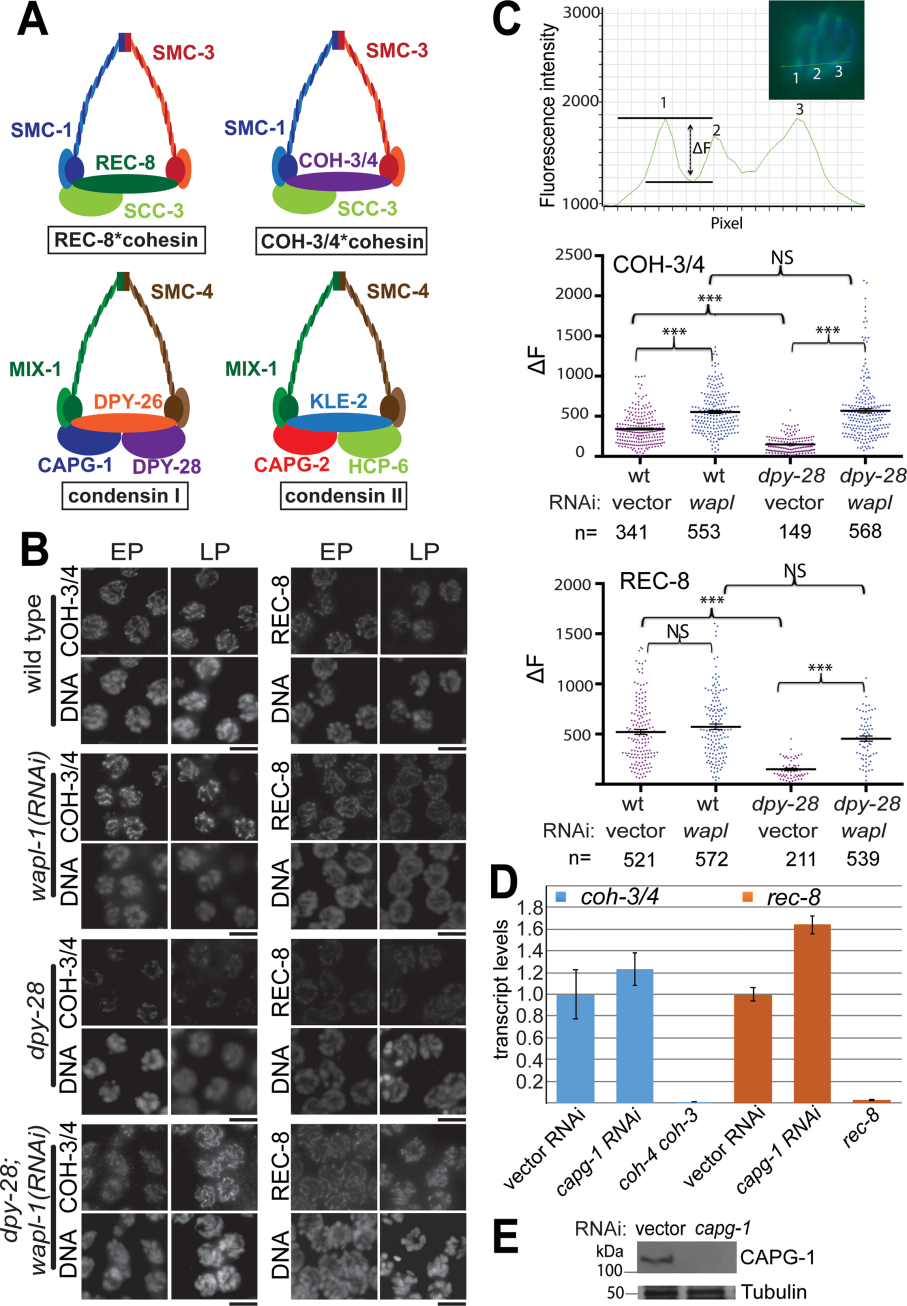
Condensin I protects cohesin from WAPL-1-mediated removal. **(A)** Cartoons of meiotic cohesin and condensin complexes. **(B)** Immunofluorescence analysis of REC-8 and COH-3/4 in early (EP) and late (LP) pachytene nuclei of *dpy-28(tm3535)* mutant and wild type males treated with empty or *wapl-1* RNAi vector. DNA was stained with DAPI. Chromosomal binding of both REC-8 and COH-3/4 is reduced in mutants but restored to nearly wild-type levels after *wapl-1* RNAi. Scale bar, 5 μm. **(C)** Fluorescence line intensity analysis to quantify chromosomal cohesin levels. Top: an intensity profile showing fluorescence intensity along a linear region of interest that crosses three chromosomes (inset; DAPI is shown in blue and REC-8 in green). Cohesin intensity is calculated as the difference (ΔF) between the maximum and minimum intensity for each cohesin track (three are shown). The scatter plots below the intensity profile show ΔF values for wild type and *dpy-28* mutant males treated with control or *wapl-1* RNAi. Mean values and SEM are indicated by long and short horizontal lines, respectively. Numbers of nuclei analyzed (n) are indicated below the graphs. Asterisks indicate levels of statistical significance by Student's t-test: * indicates p<0.05, *** indicates p<0.001, N.S. indicates not significant. **(D)** RT-qPCR analysis of *coh-3/4* and *rec-8* transcript levels in control vector or *capg-1* RNAi*-* treated *rrf-1* worms. *capg-1* RNAi did not reduce the abundance of cohesin transcripts. Transcript levels of *coh-3/4* and *rec-8* were undetectable in *coh-4 coh-3* and *rec-8* mutants, respectively. **(E)** Western blot analysis of CAPG-1 in *capg-1* RNAi*-*treated *rrf-1* worms indicates efficient depletion by RNAi. Tubulin is shown as loading control. RNAi feeding was performed for two generations. Since CAPG-1 protein and RNA are maternally loaded into oocytes, by the second generation CAPG-1 levels are reduced both in the soma and in the germline.

Mitotic cohesin consists of two Structural Maintenance of Chromosome (SMC) proteins, Smc1 and 3, a HEAT repeat domain protein, and an α-kleisin subunit that connects the head domains of the SMC subunits [1]. During meiosis, the mitotic kleisin subunit Scc1/Mcd1/Rad21 is replaced by one or more meiotic kleisins to perform meiosis-specific cohesin functions. Rec8 [2] is an essential meiotic kleisin in most organisms, but gametogenesis in many metazoans requires additional Rec8 paralogs, including COH-3 and COH-4 in *C*. *elegans* [3], RAD21L in vertebrates [4–6], and SYN3 in *Arabidopsis* [7] (Fig 1A). *C*. *elegans* COH-3 and COH-4 are highly similar and functionally redundant, and will be referred to as COH-3/4 [3]. Cohesin complexes containing different kleisin subunits load onto chromosomes using different mechanisms, have distinct localization patterns, and perform both distinct and overlapping functions [3–6,8–13].

During mitotic cell divisions, cohesin is present on chromosomes prior to DNA replication, but its association with chromatin is dynamic [14,15]. The rapid turnover of cohesin in G1 is the result of cohesin removal by an interacting protein called Wapl [14,16–18]. Following DNA replication, acetylation of Smc3 by the Eco1 acetyltransferase inhibits the Wapl-mediated cohesin release, allowing stable maintenance of SCC through G2 and early mitosis [15,19,20]. During prophase in metazoans, Wapl mediates the removal of the bulk of cohesin from chromosome arms [17,21] in a process that also requires the mitotic kinases Plk1 and Aurora B [22–24]. The remaining chromosome-bound cohesin is cleaved by separase at the metaphase-to-anaphase transition to allow separation of sister chromatids [25,26].

Cohesin dynamics are less well understood during meiosis. The mechanisms that mediate cohesin loading and SCC establishment are determined by the kleisin subunit [10]. In *C*. *elegans*, REC-8 is expressed prior to premeiotic S-phase, and REC-8 containing cohesin (REC-8*cohesin) likely becomes cohesive during DNA replication, similar to mitotic cohesin [10]. By contrast, COH-3/4 is first detected after entry into meiosis, and COH-3/4*cohesin becomes cohesive by a replication-independent mechanism that requires meiotic double strand break-initiated recombination events [10]. Recent evidence suggests that cohesin is removed in three steps during meiosis, in contrast to the two-step removal pathway seen in mitosis. First, in prophase I, a substantial portion of cohesin is removed from meiotic chromosomes in *C*. *elegans* [10,27] and mammals [5,6]. Similar to the mitotic prophase pathway, cohesin removal during meiotic prophase in mammals requires Plk1 [5]. Dependence of meiotic cohesin removal on Wapl has also been demonstrated in *Arabidopsis* [28], *C*. *elegans* [27], *S*. *cerevisiae* [29], and mice [30]. In worms, the Aurora B kinase AIR-2 [31] also plays a role. Once cohesin is removed from prophase chromosomes, its loss may be permanent, since evidence of cohesin turnover has not been observed during the prolonged meiotic prophase I, at least in the case of REC-8 in mice [32,33]. In metaphase, the association of homologs and sisters is maintained by cohesin complexes that were protected from the prophase removal process. The separation of homologs in anaphase of meiosis I requires proteolytic cleavage of a portion of the remaining cohesin by separase [34,35]; in worms, the activity of AIR-2 is also required to phosphorylate REC-8 [36,37]. Finally, cleavage of the remaining cohesin triggers separation of sister chromatids in meiosis II [33]. Different meiotic cohesins are regulated differentially by these mechanisms [10,27], suggesting the existence of additional levels of regulation.

The condensin complex is structurally related to cohesin, and is also evolutionary conserved across eukaryotes. During mitosis and meiosis, condensins promote chromosome compaction, organization, and segregation (reviewed in [38,39]). The SMC2 and SMC4 subunits of condensin form an ATPase heterodimer and associate with three regulatory proteins, called Chromosome Associated Polypeptides (CAPs), which include a kleisin subunit and two HEAT repeat containing proteins [38,39]. Metazoans have two condensin complexes (condensins I and II) with identical SMC subunits but unique sets of CAP proteins (Fig 1A). Condensin I and II have distinct localization patterns on chromosomes, suggesting a difference in function [40–44].

Previous studies have implicated condensin in the regulation of cohesin loading and activity. Condensin loading onto mitotic chromosomes coincides with the time when the bulk of cohesin is removed from chromosomes in prophase [45]. Condensin I, but not condensin II, is required for complete cohesin dissociation from the chromosomes arms in mitosis [42]. During meiosis in *S*. *cerevisiae*, condensin promotes the chromosomal localization of Cdc5 (a Plk1 homolog), which leads to cohesin phosphorylation and removal [46]. While Plk1 also plays a role in cohesin removal during mammalian meiosis [8], regulation of meiotic cohesin removal by condensin has not been reported in metazoans.

Here, we show that *C*. *elegans* condensin I protects cohesin complexes from premature removal by WAPL-1 during meiotic prophase. Disrupting condensin I function by RNAi-mediated depletion or through mutation of an essential subunit leads to reduced levels of REC-8 and COH-3/4*cohesin bound to meiotic chromosomes and causes defects in pairing and synapsis. Depletion of WAPL-1 in condensin I mutants restores both COH-3/4 and REC-8 levels on chromosomes and rescues the pairing defects seen in mid and late pachytene. Previous studies suggested that WAPL-1 preferentially targets COH-3/4*cohesin for removal [27]. Our results indicate that in condensin I mutants, WAPL-1 prematurely removes both REC-8 and COH-3/4*cohesins, revealing a previously unrecognized function of condensin in promoting the stable binding of cohesin to chromosomes during gametogenesis.

## Results

### Condensin I promotes the chromosomal association of meiotic cohesins

Because condensins I and II share the same SMC subunits but differ in their CAP subunits, we disrupted a CAP subunit of each complex to determine whether either condensin influences meiotic cohesin dynamics. Condensin I function was severely compromised by *dpy-28(tm3535)*, a likely null allele of the gene encoding the *C*. *elegans* CAP-D2 ortholog, or by depletion of CAPG-1 by RNAi. Condensin II function was disrupted by RNAi depletion of the CAPG-2 subunit. Because DPY-28, CAPG-1, and CAPG-2 are required for somatic functions that are essential for embryonic and larval development, we utilized strategies to minimize somatic defects while still efficiently disrupting condensin function in the germline (see Methods and below).

DPY-28 and CAPG-1 are components of two condensin complexes: condensin I and the dosage compensation-specific complex condensin I^DC^ [41,47]. Dosage compensation reduces the expression of the two hermaphrodite X chromosomes to equalize gene dose with that of the single X chromosome in males. Dosage compensation is essential in hermaphrodites, and hermaphrodites lacking functional condensin I^DC^ arrest before reaching reproductive maturity. Dosage compensation is not implemented in males, which therefore remain viable in the absence of functional condensin I^DC^, allowing the study of dosage compensation-independent roles of DPY-28 and CAPG-1.

To determine whether condensin I regulates meiotic cohesin, we first examined the levels of REC-8 and COH-3/4 on meiotic chromosomes of *dpy-28(tm3535)* mutant males produced by maternally rescued, homozygous mutant hermaphrodites (hereafter, *dpy-28* males; see Methods). Because these males are the grandchildren of the last generation to carry a wild-type allele of *dpy-28*, it is expected that they completely lack condensin I function. *dpy-28* males appear superficially wild type and have largely normal germlines. In wild type males, REC-8 was detected in mitotically proliferating nuclei at the distal tip of the gonad and in all meiotic nuclei, while COH-3/4 was first detected in the transition zone, similar to the patterns previously reported [10,48]. Similar REC-8 and COH-3/4 patterns were observed in *dpy-28* male germlines; however, the levels of both cohesins appeared diminished on meiotic chromosomes (Fig 1B). REC-8 levels were unchanged in mitotic nuclei, but were diminished at entry into transition zone in mutants. COH-3/4 remained undetectable in mitotic nuclei, but from the transition zone on, COH-3/4 levels were also reduced compared to wild type (S1A Fig). Despite these changes in staining intensities in transition zone and pachytene nuclei, at later stages in meiosis (metaphase I), wild type and mutant germlines appeared similar (S1B Fig), indicating that the cohesin complexes remaining on chromosomes at this stage are regulated similarly in wild type and condensin I mutant germlines (see discussion).

To quantify cohesin levels in pachytene, we performed line intensity analysis across REC-8 and COH-3/4-stained pachytene nuclei. We measured the difference in fluorescence intensity between chromosome axes and interchromosomal regions, similar to a previous analysis [27] (Fig 1C). Our measurements clearly showed that REC-8 and COH-3/4 levels are reduced on chromosomes in *dpy-28* mutant male germlines (p<0.001, t-test).

Next we assessed whether cohesin levels are similarly affected in hermaphrodites using RNAi depletion of condensin I subunit CAPG-1 (see Methods). To minimize phenotypes resulting from defects in somatic dosage compensation, we performed this analysis in *rrf-1(ok589)* hermaphrodites. The efficiency of RNAi is reduced in the soma of *rrf-1* mutants [49], allowing us to study gene function in the germline while minimizing somatic defects. For all *capg-1(RNAi*) experiments in this study, we monitored depletion efficiency by western blot analysis. A typical blot is shown on Fig 1E. In *capg-1*(*RNAi)* hermaphrodites at pachytene, COH-3/4 and REC-8 were diminished compared to control animals treated with empty vector (S2A Fig). Although the levels of REC-8 and COH-3/4 are markedly reduced in *dpy-28* mutant males and *capg-1*(*RNAi*) hermaphrodites, both kleisins still associate with meiotic chromosomes, as the staining intensities were clearly higher than those in *rec-8(ok978)* or *coh-4(tm1857) coh-3(gk112)* null mutants (S2B Fig). The reduction in staining was milder than in *dpy-28* mutant males, perhaps as a consequence of incomplete depletion; nevertheless, these results demonstrate that chromosomal association of cohesin is reduced upon loss of condensin I function in either sex. At diakinesis, distributions of COH-3/4 and REC-8 were similar to controls, suggesting that condensin I regulates meiotic cohesins earlier in prophase (S2C Fig).

To investigate whether condensin II also influences the chromosomal localization of cohesin, we used *rrf-1* hermaphrodites depleted of condensin II subunit CAPG-2 using RNAi. Since condensin II plays a more dominant role than condensin I in *C*. *elegans* [41], prolonged exposure to condensin II RNAi is lethal. We therefore employed a shortened, one-generation feeding protocol (see Methods). Under these conditions, we found no difference in cohesin localization between *capg-2*(*RNAi)* and control hermaphrodites (S2D Fig). While we cannot exclude the possibility that more complete depletion of condensin II would affect meiotic cohesin loading, we conclude that condensin I has a more pronounced role in the regulation of meiotic cohesin than does condensin II. For the rest of this study, we concentrated on analyzing defects in condensin I mutants.

One possible explanation for the diminished association of cohesin with meiotic chromosomes following disruption of condensin I is a decrease in the overall abundance of one or more cohesin subunits. Because condensin influences transcription in a number of organisms, and condensin I^DC^ regulates X chromosome-wide transcription to implement dosage compensation in *C*. *elegans* (reviewed in [50]), we tested whether condensin I disruption affected the abundance of transcripts encoding meiosis-specific cohesin subunits. The *coh-3/4* primers used in this analysis amplify both *coh-3* and *coh-4* transcripts. RT-qPCR analysis demonstrated that *rec-8* and *coh-3/4* transcript levels were not reduced in *capg-1* RNAi-treated *rrf-1* worms compared to empty vector-treated controls (Fig 1D). As expected, *rec-8* and *coh-3/4* transcripts were undetectable in *rec-8(ok978)* and *coh-4(tm1857) coh-3(gk112)* mutants, respectively. These results suggest that the reduced chromosomal association of REC-8 and COH-3/4 in gonadal nuclei of condensin I-disrupted animals are not the result of reduced transcription of kleisin genes.

### Condensin I is required for normal homolog pairing and sister chromatid cohesion

**Fig 2:**
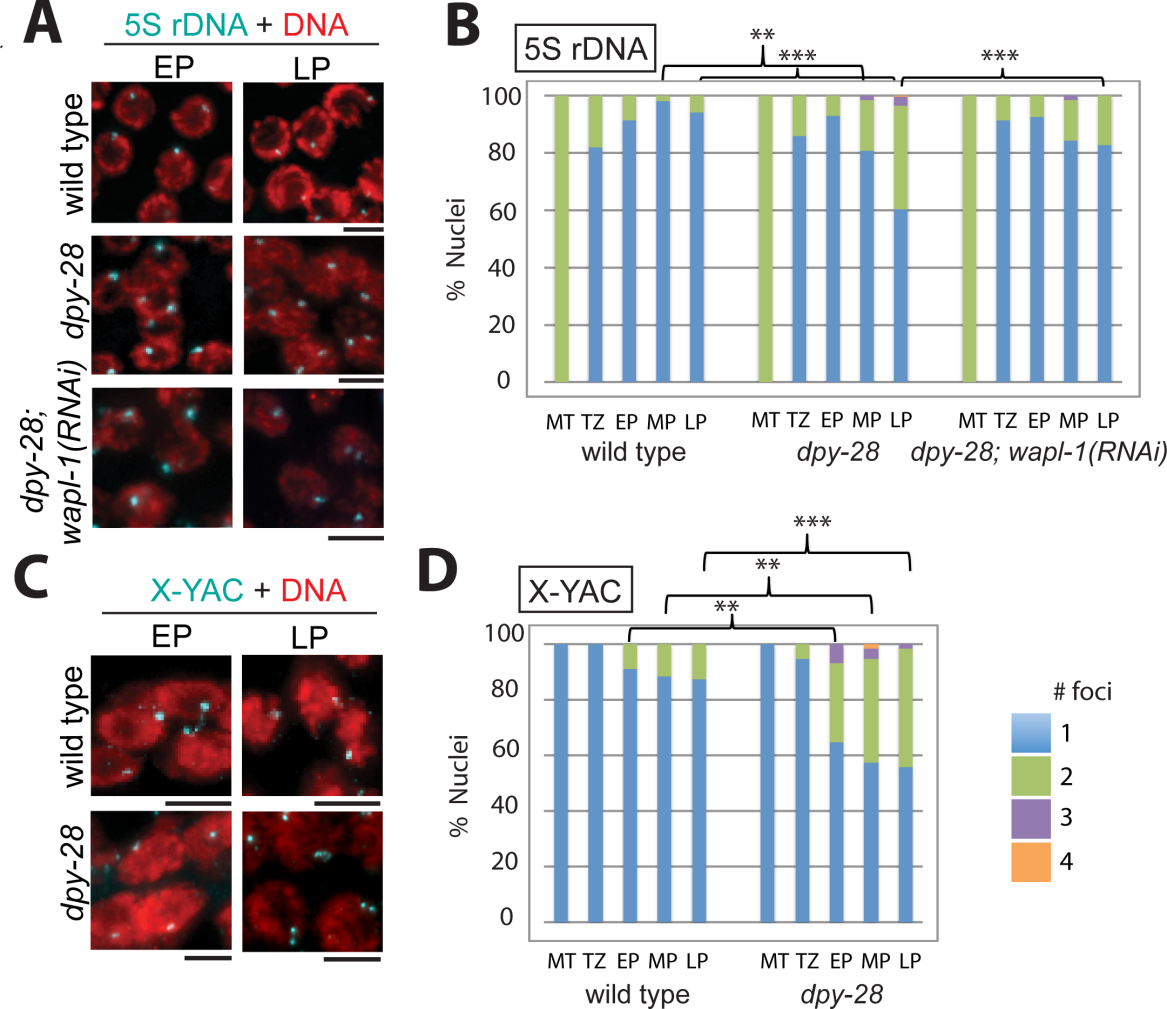
Defects in homolog pairing and sister chromatid cohesion in condensin I mutants. **(A)** 5S rDNA FISH analysis of early and late pachytene nuclei in the germline in wild type and *dpy-28(tm3535)* males, as well as in mutants with *wapl-1(RNAi)*. Nuclei with two foci, indicating unlinked homologs, are more frequently seen in *dpy-28* mutants, and this defect is rescued by *wapl-1(RNAi)*. Scale bar, 5 μm. **(B)** Graph showing the percentage of nuclei with paired 5S rDNA signal in the mitotic tip (MT), transition zone (TZ), early pachytene (EP), mid-pachytene (MP) and late pachytene (LP). **(C)** FISH analysis using an X-liked YAC probe of early and late pachytene nuclei in the germline in wild type and in *dpy-28(tm3535)* males. Nuclei with two foci, indicating unlinked sister chromatids, are more frequently seen in *dpy-28* mutants. Scale bar, 5 μm. **(D)** Graph showing the percentage of nuclei with the given numbers of X signals. In (B) and (D) blue indicates 1 focus (sister chromatid cohesion intact), green indicates 2, purple indicates 3, and orange indicates 4 foci per nucleus. Asterisks indicate statistical significance by two-tailed Fisher's exact test comparing the numbers of nuclei with 1 focus versus the numbers of nuclei with 2 or more foci. ** indicates p<0.01 ***, p<0.001. Numbers of nuclei analyzed and p values are shown in S1 Table.

To determine whether the reduction in chromosomally-bound REC-8 and COH-3/4*cohesin we observed following disruption of condensin I has functional consequences on meiotic progression, we used fluorescence in situ hybridization (FISH) to monitor pairing of homologous chromosomes and cohesion between sister chromatids (Fig 2). Homolog pairing, stabilized by synapsis, is cohesin dependent and facilitates the formation of interhomolog crossovers in *C*. *elegans* [51–54]. In gonads hybridized with a 5S rDNA FISH probe, detection of a single fluorescent focus per nucleus indicates that the two homologs of chromosome V are paired and sister chromatids are held tightly together by SCC. Two foci separated by less than 0.75 μm are also interpreted as paired [53]. Two foci separated by greater than 0.75 μm are considered unpaired, and the presence of three or more FISH foci is evidence of SCC defects in addition to pairing defects.

In wild type males, pairing was first detected in the transition zone, and homologs remained paired throughout pachytene. However, in *dpy-28* males, pairing appeared normal in early pachytene, but it was not maintained. By late pachytene, about 40% of nuclei in *dpy-28* gonads had two or more distinguishable foci, compared to only 5% of nuclei in wild type (p<0.001, two-tailed Fisher's exact test). Pairing defects were observed as early as mid-pachytene and persisted to late pachytene (p<0.01) (Fig 2A and 2B).

To examine linkages between sister chromatids, we used an X-linked yeast artificial chromosome (YAC) as a FISH probe. Because males possess a single X chromosome, detection of two discrete FISH foci is indicative of sister separation. Again, when compared to wild type males, *dpy-28* mutants had a higher frequency of unlinked sister chromatids in all stages of pachytene (p<0.01, Fisher's exact test) (Fig 2C and 2D). By late pachytene, two foci were detected in nearly 50% of nuclei in *dpy-28* mutants. The frequency of detached X chromosomes (Fig 2D) was greater than the observed defects for chromosome V (Fig 2B), suggesting that linkages between sister chromatids of the X chromosomes are more severely affected by condensin I disruption than linkages between the sister chromatids of autosomes.

### Condensin I is required for normal assembly of the synaptonemal complex

**Fig 3:**
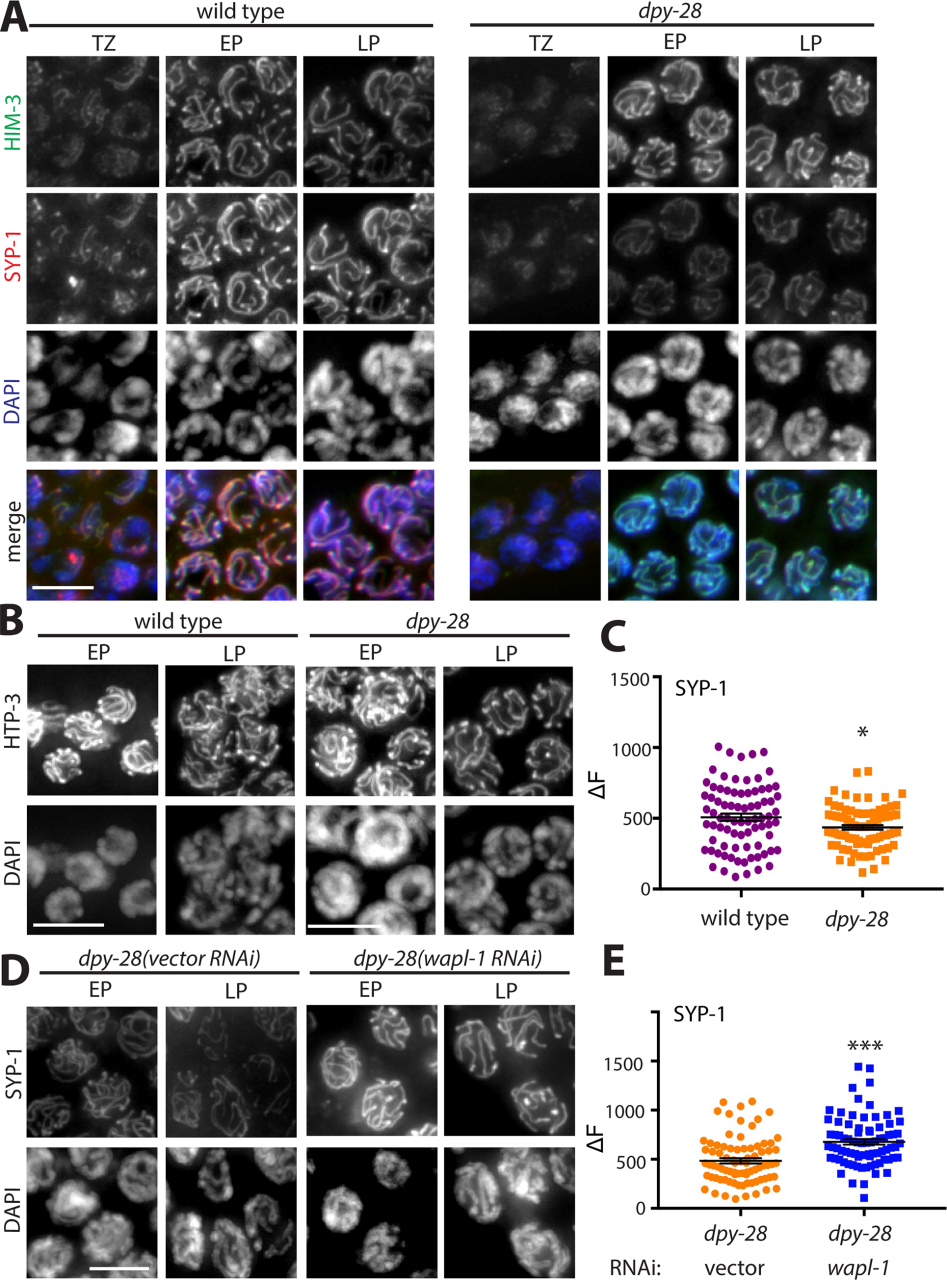
SC assembly defects in condensin I mutants. **(A)** Immunofluorescence analysis of axial element HIM-3 (green) and central elements SYP-1 (red) in wild type and *dpy-28(tm3535)* mutant male gonads in the transition zone (TZ), early pachytene (EP), and late pachytene (LP). DNA is stained with DAPI (blue) **(B)** Immunofluorescence analysis of axial element protein HTP-3. HTP-3 and HIM-3 staining in mutants appears indistinguishable from wild type, but SYP-1 levels are reduced in mutants. **(C)** Fluorescence line intensity quantification shows that chromosomal levels of SYP-1 are reduced in *dpy-28* mutants (n = 80, p = 0.0209, Student’s t-test). **(D)** Immunoflurescence analysis of SYP-1 in *dpy-28(tm3535)* mutants treated with control vector or *wapl-1* RNAi. **(E)** Line intensity quantification shows that *wapl-1* RNAi leads to an increase in chromosomal levels of SYP-1 in *dpy-28* mutants (n = 80, p<0.001, Student’s t-test). Error bars indicate SEM. Scale bar, 5 μm.

The SC is a tripartite structure that forms between homologous chromosomes and facilitates meiotic crossover formation. SC assembly occurs in two steps: First, linear structures called axial elements (AEs) assemble along the length of each meiotic chromosome. Next, central region proteins crosslink homologous AEs during synapsis. Meiotic cohesin is required for SC assembly in most eukaryotes examined [2,3,9,12,55,56]. To determine whether mutations in condensin I disrupt SC assembly, we stained wild type and *dpy-28* mutant males with antibodies specific to the AE components HTP-3 and HIM-3 and to the central region protein SYP-1. In pachytene nuclei of wild type males, all three proteins localize along the length of chromosomes. In *dpy-28* mutants, HTP-3 and HIM-3 appear normal, but SYP-1 levels are reduced (Fig 3A and 3B). Quantification of SYP-1 levels is shown on Fig 3C. These results suggest that the diminished levels of cohesin are sufficient for AE assembly, but not for the loading of normal levels of SYP-1 between homologous AEs. Interestingly, *lab-1* mutants have a similar phenotype [31]. LAB-1 promotes establishment of sister chromatid cohesion in meiotic prophase I by antagonizing the Aurora B kinase AIR-2 [31].

To test whether disrupting condensin II has similar effects, we investigated SC assembly in gonads depleted of condensin II subunit CAPG-2 by RNAi. Localization of SC components HTP-3 and SYP-1 were unaltered after condensin II depletion (S2D Fig). This result is consistent with the unchanged cohesin levels in these germlines.

### Condensin I dysfunction increases the width of the synaptonemal complex

**Fig 4:**
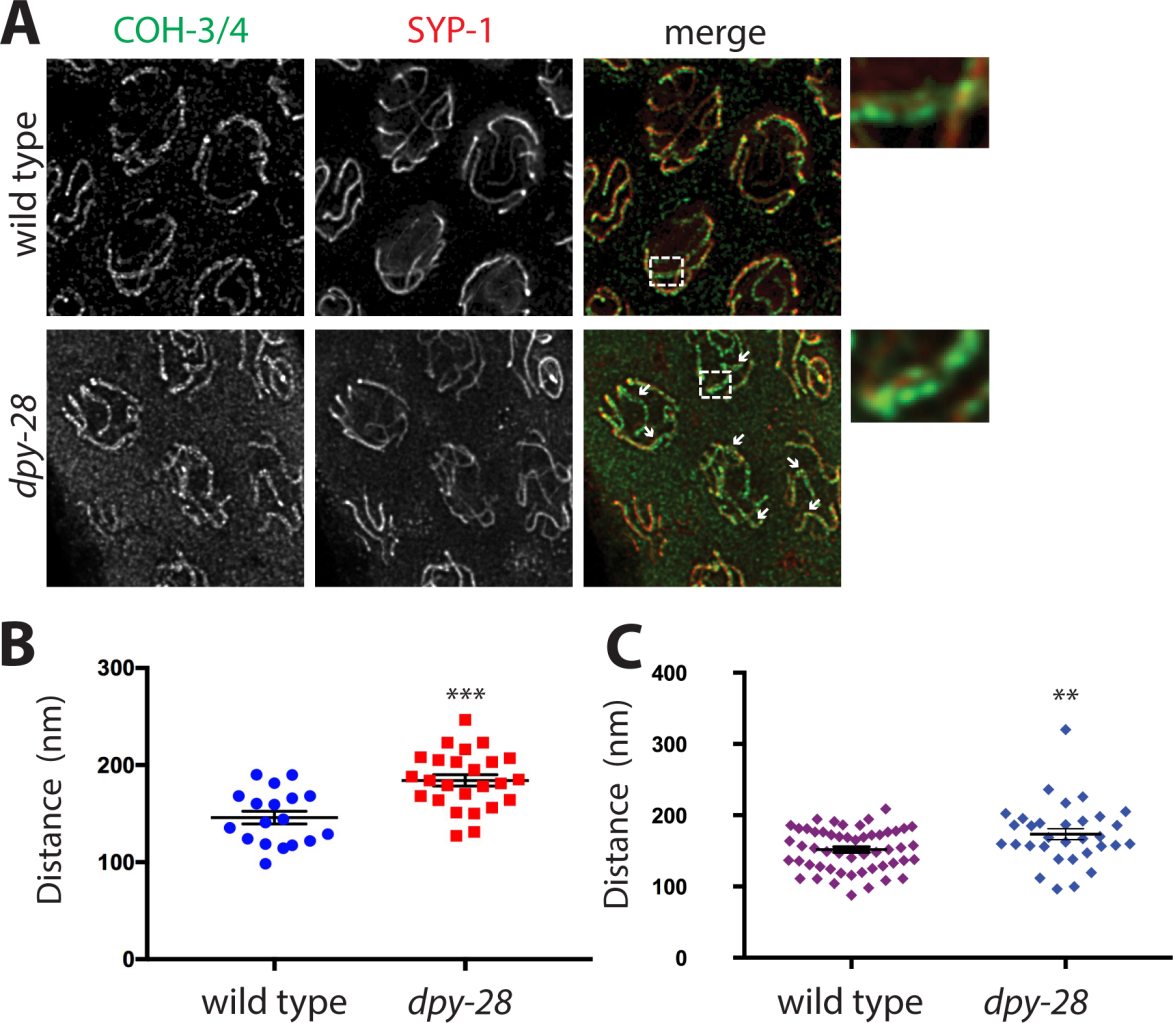
Defects in axial element separation condensin I mutants. **(A)** STED microscopy images of cohesin (COH-3/4, green) and central element (SYP-1, red) localization on pachytene nuclei in wild type and *dpy-28(tm3535)* mutant males. Enlarged region is shown on the right. In both genotypes, two parallel tracks of cohesin along chromosomes can be resolved with a single SYP-1 track in between. Condensin I mutants show greater distances between the COH-3/4 tracks (arrows), indicating defects in chromosome axis formation. Because both COH-3/4 and SYP-1 signal intensities are reduced in *dpy-28* mutants, we increased the laser power and detector gain to collect data, which resulted in higher levels of background in the mutants. **(B)** Distance measurements between COH-3/4 tracks in wild type (n = 18), and *dpy-28* mutants (n = 25) at locations chosen randomly along chromosomes. **(C)** Distance measurements between COH-3/4 tracks in wild type (n = 52) and *dpy-28* mutants (n = 32) at locations with a clear SYP-1 signal between the tracks. Asterisks indicate statistical significance by two-tailed, unpaired Student's t-test (*** indicates p<0.001, ** indicates p<0.01). Error bars indicate SEM.

Because SYP-1 staining was significantly reduced in *dpy-28* mutants, we asked whether disrupting condensin I alters the structure of the SC. Previous electron microscopy measurements indicated that in wild-type worms, the distance between paired AEs is approximately 100 nm [57–60]. Using conventional fluorescence microscopy, cohesin, AE proteins, and SC central region proteins all appear to co-localize in a single track between homologs. Using stimulated emission depletion (STED) microscopy, we could resolve two parallel tracks of COH-3/4 flanking a single track of SYP-1 in pachytene nuclei of males, comparable to previous analysis of the SC by superresolution microscopy [48,61,62] (Fig 4A). Parallel tracks of COH-3/4 were observed in wild type and mutant samples; however, the tracks were further apart in *dpy-28* mutants. For quantification, we selected two to five regions in each nucleus at random positions along chromosomes with two clearly resolved COH-3/4 tracks. We then measured the distances between COH-3/4 tracks in these regions. We included mid-to-late pachytene nuclei in our analysis. In wild type worms, the average distance between tracks was 146 nanometers, slightly larger than previous measurements of the distance between AE protein tracks (100–120 nm) [57,61,62], but very close to the measured distance between tracks of COH-3, REC-8 or the head domains of SMC cohesin subunits (about 140 nm) [62]. In *dpy-28* mutants, the width of the SC was significantly increased to an average of 184 nanometers (Fig 4B, p = 0.000102, Student's t-test). Increased distances between homologs are seen both at the ends of chromosomes and in the middle regions, as indicated by the arrows in Fig 4A. Next, to ensure that we were analyzing chromosomal regions where the SC is in fact assembled, we limited this analysis to COH-3/4 tracks with clear SYP-1 staining in between. In this analysis, the difference between wild type (152 nm) and mutant (174 nm) diminished, but was still statistically significant (p = 0.00692, Student’s t-test) (Fig 4C). This difference remained statistically significant even after removing the one outlier in the mutant data set (p = 0.0181, Student’s t-test). Taken together, our results suggest that the reduced levels of cohesin in condensin I mutants interfere with the recruitment of SC central region proteins like SYP-1, which results in an increased width of the SC as measured by the distance between tracks of COH-3/4. However, the reduced levels of condensin I and/or cohesin activity may also directly affect chromosome structure in such a way that the distance between cohesin tracks of paired chromosomes increases. Alternatively, cohesin complexes closest to the central region may depend on condensin I to a greater degree than cohesin complexes farther away. Higher resolution studies will be needed to distinguish between these possibilities.

### Condensin I influences the steady-state level of meiotic DSBs

In *C*. *elegans* hermaphrodites, condensin I limits double strand DNA break (DSB) number and regulates DSB distribution, and thereby influences the number and distribution of crossover recombination events [47,60]. To determine whether this role of condensin I is conserved during spermatogenesis, we monitored DSB formation and repair in wild type and *dpy-28* mutant male gonads stained with antibodies that recognize RAD-51, a RecA homolog that marks recombination intermediates [52]. As in hermaphrodites, we observed a significant increase in the number and intensity of RAD-51 foci in early and mid-pachytene nuclei of mutant males (S3A and S3B Fig). By late pachytene, the numbers of foci decreased, as in wild type, indicating that these recombination intermediates are eventually resolved. We speculate that the increase in RAD-51 foci may be a consequence of condensin I disruption leading to an increase in the number of DSBs produced, as shown previously in condensin I deficient hermaphrodites [47]. Alternatively, decrease in cohesin levels and disruption of SC structure may interfere with repair.

Defective germline nuclei in *C*. *elegans* hermaphrodites are eliminated by apoptosis [63], and this process can be monitored by observing expression of CED-1::GFP, a protein which marks the cells engulfing the apoptotic germ cell [64]. Using this method, we observed an increase in apoptosis in the germline, suggesting that some of the resulting defective nuclei may be eliminated in condensin I-deficient hermaphrodite gonads (S3C Fig).

### Condensin I regulates meiotic functions of both REC-8* and COH-3/4*cohesin

**Fig 5:**
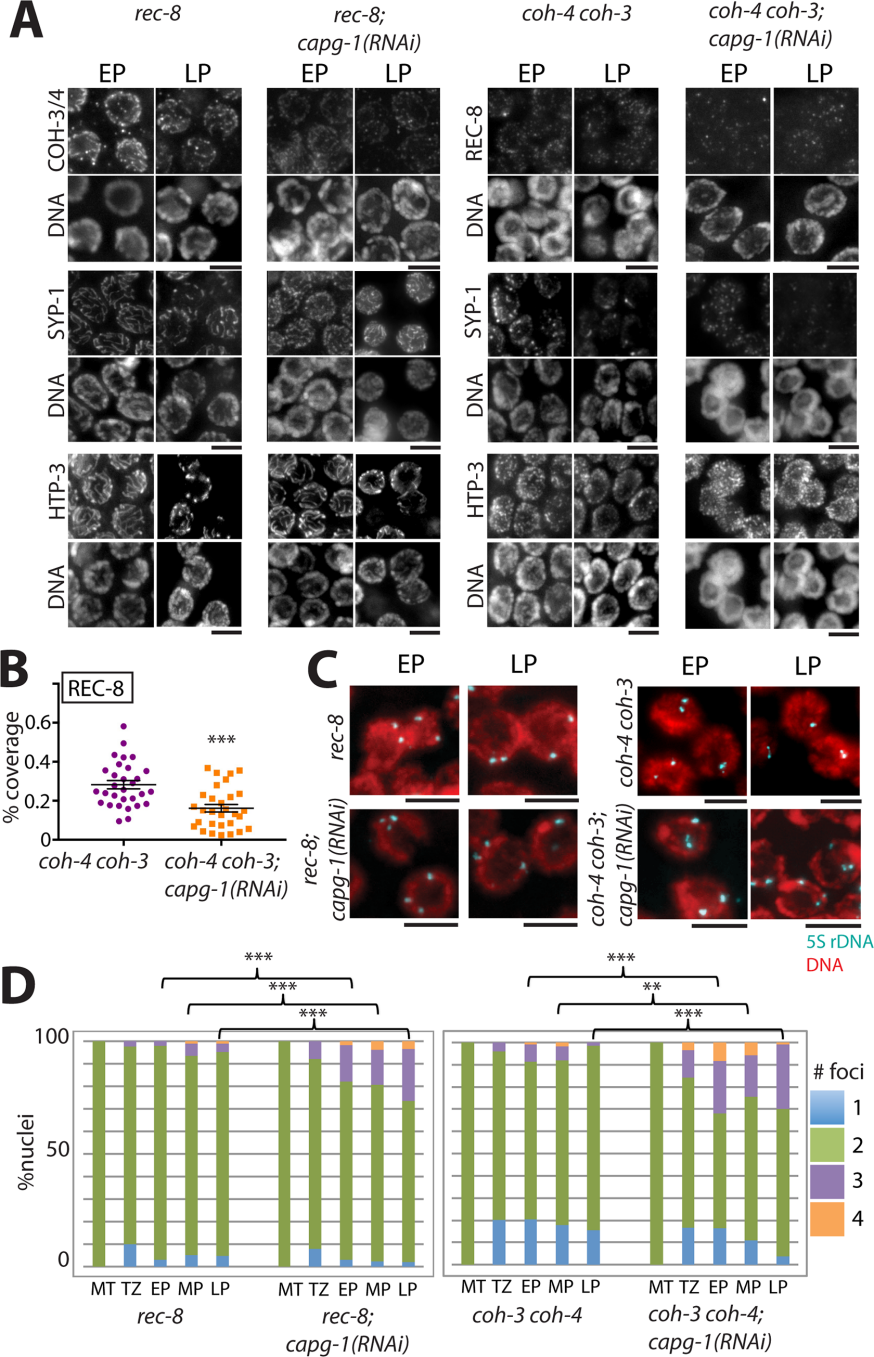
Condensin I affects both meiotic cohesins. **(A)** Immunofluorescence images of REC-8 or COH-3/4, as well as SC components SYP-1 and HTP-3 in *rec-8* or *coh-4 coh-3* mutants treated with control vector or *capg-1* RNAi in early pachytene (EP) or late pachytene (LP). Levels of both cohesins are reduced, and SYP-1 levels are reduced in *coh-4 coh-3* mutants after condensin I depletion. Scale bar, 5 μm. **(B)** Quantification of REC-8 signal on chromosomes of *coh-4 coh-3* mutants. The proportion of DNA (DAPI) signal overlapping with REC-8 signal is significantly decreased in *capg-1*(*RNAi)* (p<0.001, Student’s t-test). **(C)** 5S rDNA FISH analysis of early and late pachytene nuclei in *rec-8* or *coh-4 coh-3* mutants with control vector or *capg-1(RNAi)*. **(D)** Graphs showing the percentage of nuclei with 1, 2, 3, or 4, 5S rDNA signals. Asterisks indicate statistical significance using Chi square test with three categories (1 focus, 2 foci, 3 or 4 foci). ** indicates p<0.01, *** indicates p<0.001. Numbers of nuclei analyzed and p values are shown in S1 Table.

COH-3/4*cohesin and REC-8*cohesin are loaded onto meiotic chromosomes at distinct times using different mechanisms [10]. To analyze the effects of condensin I on each cohesin complex independently, we disrupted condensin I by *capg-1* RNAi in hermaphrodites lacking either REC-8 or COH-3/4. In *rec-8* mutants (containing only COH-3/4*cohesin), COH-3/4, HTP-3, and SYP-1 all appear in long, continuous tracks extending the length of meiotic chromosomes (Fig 5A) [3,10]. Depleting CAPG-1 by RNAi in *rec-8* mutants led to reduced COH-3/4 signal; however, SYP-1 and HTP-3 levels were comparable to those observed in control *rec-8* animals treated with empty vector, suggesting that the remaining residual levels of COH-3/4 are sufficient for SC assembly. Note that RNAi depletion of CAPG-1 likely does not reduce condensin I function to the same degree as the *dpy-28* mutation, explaining why we observed reductions in SYP-1 levels in *dpy-28* mutants, but not after CAPG-1 RNAi. In *coh-4 coh-3* double mutants (only REC-8*cohesin is present), REC-8, SYP-1, and HTP-3 are all detectable on chromosomes in short stretches rather than continuous linear structures (Fig 5A) [3,10]. *capg-1* RNAi in *coh-4 coh-3* hermaphrodites further reduced the chromosomal association of REC-8. Fluorescence intensity of the short REC-8 stretches appeared unchanged; however, the proportion of the DNA covered by REC-8 signal significantly diminished (Fig 5B) (p<0.001). *capg-*1 RNAi also diminished chromosomal SYP-1 levels, but HTP-3 levels were unaffected (Fig 5A). As in *coh-4 coh-3* double mutants, SYP-1 formed short, fragmented stretches on chromosomes in pachytene nuclei of *capg-1*(*RNAi*); *coh-4 coh-3* animals. This phenotype was distinct from that resulting from complete failure of SC assembly, as occurs in *htp-3* mutants in which SYP-1 is present in nuclear aggregates (S4 Fig, [55]). These results indicate that the limited quantity of REC-8 remaining on the chromosomes of *capg-1*(*RNAi*); *coh-4 coh-3* animals is sufficient for SC components to associate with chromosomes, and that SYP-1 is more sensitive to reductions in REC-8 levels than is HTP-3.

To determine whether the reduction of chromosomally-bound cohesin we observed after condensin I disruption in *rec-8* single mutants and *coh-4 coh-3* double mutants led to functional consequences, we performed 5S rDNA FISH analysis. *coh-4 coh-3* mutants treated with empty RNAi vector had two foci in 70–80% of transition zone and pachytene nuclei, indicating severe defects in homolog pairing consistent with the severely disrupted organization of HTP-3 and SYP-1 in these mutants [3] (Fig 5C and 5D). *rec-8* mutants raised on control vector also had two foci in ~90% of meiotic nuclei, suggesting that the robust, linear SC detected in these mutants by HTP-3 and SYP-1 staining forms between nonhomologous chromosomes or sister chromatids. This is consistent with a previous study that examined pairing of chromosome V in *rec-8* by a different method [10], but a recent study found that X chromosomes of *rec-8* mutants undergo largely homologous pairing [62]. Thus, the absence of REC-8 may have differential effects on autosomes and sex chromosomes in *C*. *elegans* hermaphrodites.

The presence of two 5S rDNA foci in most nuclei of *rec-8* and *coh-4 coh-3* worms treated with control vector RNAi indicates that SCC remains largely intact. However, *rec-8* and *coh-4 coh-3* mutants treated with *capg-1* RNAi had a greatly increased frequency of nuclei with three or four foci, indicating that disruption of condensin I enhanced the defects in sister chromatid cohesion (Fig 5D; p<0.01 in all stages of pachytene, Chi square test). Moreover, the frequency of detached sister chromatids in *capg-1*(*RNAi*); *rec-8* and *capg-1*(*RNAi*); *coh-4 coh-3* animals was much higher than that observed in *dpy-28* mutant males with wild-type alleles of *rec-8*, *coh-3*, and *coh-4*. Thus, depletion of condensin I disrupts SCC mediated by both REC-8 and COH-3/4*cohesin.

**Fig 6:**
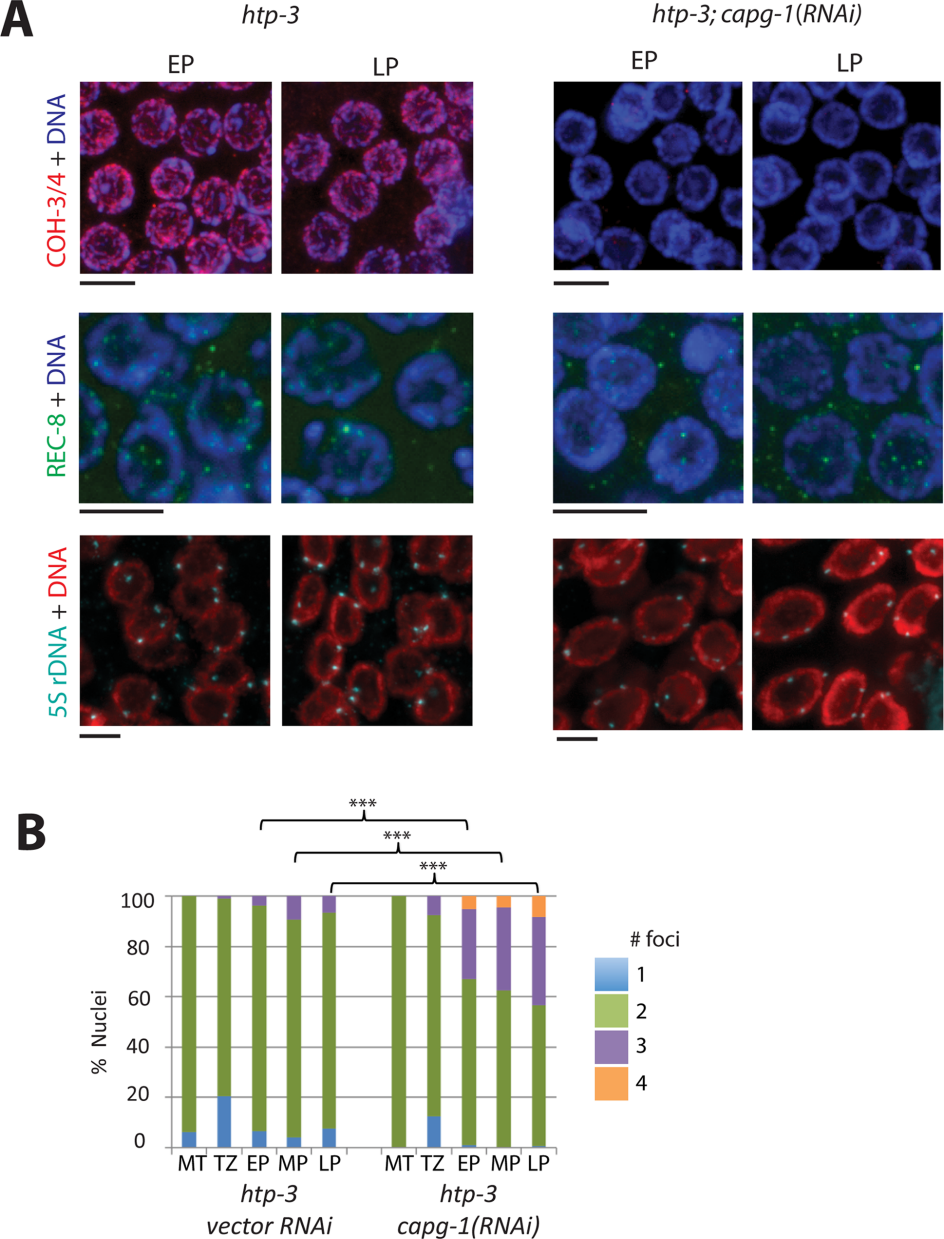
Condensin I promotes SCC mediated by COH-3/4*cohesin in *htp-3* mutants. **(A)** (top) Immunofluorescence images of COH-3/4 (red) in *htp-3* mutants and in *htp-3* mutants treated with *capg-1* RNAi. Levels and chromosomal association of COH-3/4 are normal in *htp-3* mutants, but are severely reduced in *htp-3; capg-1(RNAi)*. (middle) Immunofluorescence images of REC-8 (green) in *htp-3* mutants and in *htp-3* mutants treated with *capg-1* RNAi. REC-8 levels are undetectable in both backgrounds. (bottom) 5S rDNA FISH analysis in *htp-3* mutants and in *htp-3* mutants treated with *capg-1* RNAi. Scale bar, 5 μm. **(B)** Quantification of 5S rDNA data in (A). In *htp-3* mutants many pachytene nuclei exhibit two 5S rDNA foci, indicating defects in homolog pairing. In *htp-3; capg-1(RNAi)*, a significant number of nuclei have 3 or 4 foci, indicating defects in sister chromatid cohesion. Asterisks indicate statistical significance using Chi square test with three categories (1 focus, 2 foci, 3 or 4 foci) in pachytene nuclei. *** indicates p<0.001. Numbers of nuclei analyzed and p values are shown in S1 Table.

These conclusions were confirmed when analyzing *htp-3* mutants. The AE protein HTP-3 is required for chromosomal loading of REC-8*cohesin, but not COH-3/4*cohesin [10]. As reported previously, only residual amounts of REC-8 were detected on chromosomes of *htp-3* mutants, while COH-3/4 was relatively unaffected (Fig 6A) [10]. When we treated *htp-3* mutants with *capg-1* RNAi, chromosomal COH-3/4 levels were severely reduced, similar to the results obtained in *rec-8* mutants. 5S rDNA FISH analysis also indicated that *capg-1*(*RNAi*) exacerbates the sister chromatid cohesion defects of *htp-3* mutants (Fig 6A and 6B). The majority of germline nuclei in *htp-3* mutants have two 5S rDNA foci, but *capg-1* RNAi-treated mutants have a large portion of nuclei with 3 or 4 foci, indicating the loss of cohesin-dependent linkages between sister chromatids (Fig 6B). Differences were highly statistically significant at all stages of pachytene (p<0.001, Chi-square test). Thus, the chromosomal association of cohesin and the ability of cohesin to mediate sister chromatid cohesion are affected by loss of condensin I in all genetic backgrounds tested.

### Condensin I depletion does not increase the frequency of cohesion defects in *rec-8; coh-4 coh-3* triple mutants

**Fig 7:**
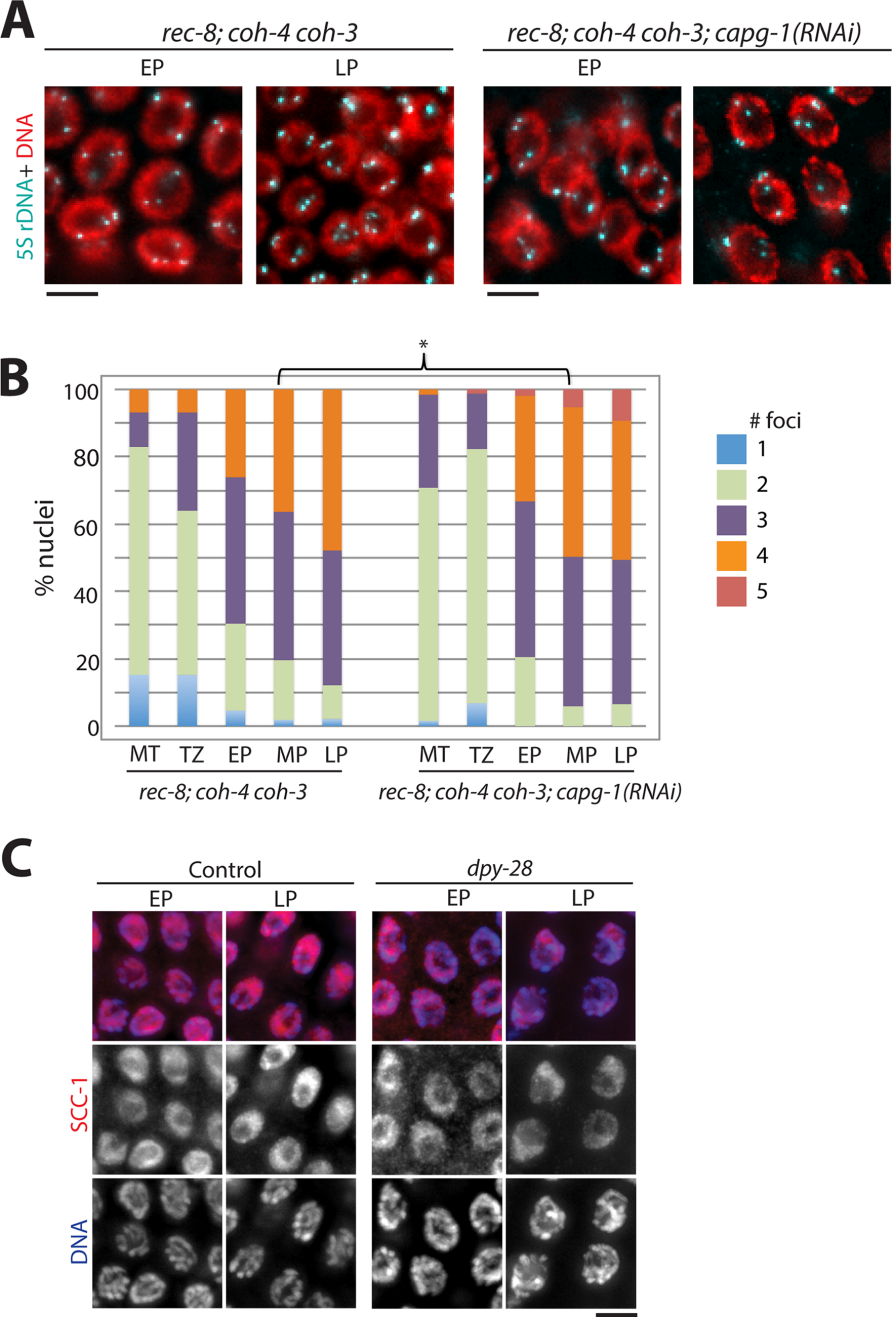
Condensin I does not affect chromosomal linkages independent of meiotic cohesins. **(A)** 5S rDNA FISH analysis in *rec-8; coh-4 coh-3* mutants with or without *capg-1*(*RNAi*). **(B)** Quantification of data in (A). *capg-1* RNAi did not cause further disruption in chromosomal linkages. Chi square test with four categories (1 focus, 2 foci, 3 foci, 4 or 5 foci) of pachytene stages only revealed weak significance in mid-pachytene. * indicates 0.01<p<0.05. Numbers of nuclei analyzed and p values for all stages are shown in S1 Table. **(C)** Immunofluorescence analysis of SCC-1 (red) localization in wild type and *dpy-28(tm3535)* m-z- male gonads in early pachytene (EP) and late pachytene (LP). SCC-1 localization did not change in mutants. Scale bar, 5 μm.

The increased sister separation we observed following condensin I disruption in wild-type animals and *rec-8* and *coh-4 coh-3* mutants could result entirely from the reduced chromosomal association of REC-8 and COH-3/4*cohesin in *dpy-28* mutant males and *capg-1*(*RNAi*) hermaphrodites. Alternatively, disrupting condensin I function could compromise REC-8 and COH-3/4-independent linkages between sister chromatids. We therefore used 5S rDNA FISH to examine sister associations in *rec-8; coh-4 coh-3* triple mutants treated with *capg-1* RNAi. Nearly 50% of mid-to-late pachytene nuclei of kleisin triple mutants had four 5S rDNA foci regardless of whether they were treated with *capg-1* or control RNAi vectors (Fig 7A and 7B). Chi square analysis of pachytene stages indicated that differences between control and *capg-1*(*RNAi*) samples were not significant in early pachytene (p = 0.074) and late pachytene (p = 0.058), and only moderately significant in mid-pachytene (p = 0.019). Thus, condensin I likely strengthens SCC by promoting the association of REC-8 and COH-3/4-containing cohesins with mitotic chromosomes.

A recent study showed that SCC-1, a kleisin subunit of cohesin previously thought to function only during mitosis, localizes on chromosomes and aids cohesion in early meiosis [10]. SCC-1 localization was not altered in *dpy-28* mutant germlines compared to wild type (Fig 7C), consistent with the interpretation that condensin I impacts REC-8 and COH-3/4*cohesins, but not cohesion mediated by other factors.

### Condensin I protects meiotic cohesin from WAPL-1-mediated removal

The data described above demonstrate that disruption of condensin I leads to decreased levels of REC-8 and COH-3/4 on meiotic chromosomes and, consequently, weakened SCC and defects in the structure of the SC. Based on the known mechanisms that regulate cohesin levels during meiosis in *C*. *elegans* and other organisms, we formulated two hypotheses regarding how condensin I might promote the chromosomal association of meiotic cohesin. In the first model, condensin I counteracts the Aurora B kinase AIR-2. In the second model, condensin I acts in opposition to Wapl. AIR-2 and Wapl have both been shown to trigger the removal of cohesin from meiotic chromosomes in *C*. *elegans* [27,31,36,37].

**Fig 8:**
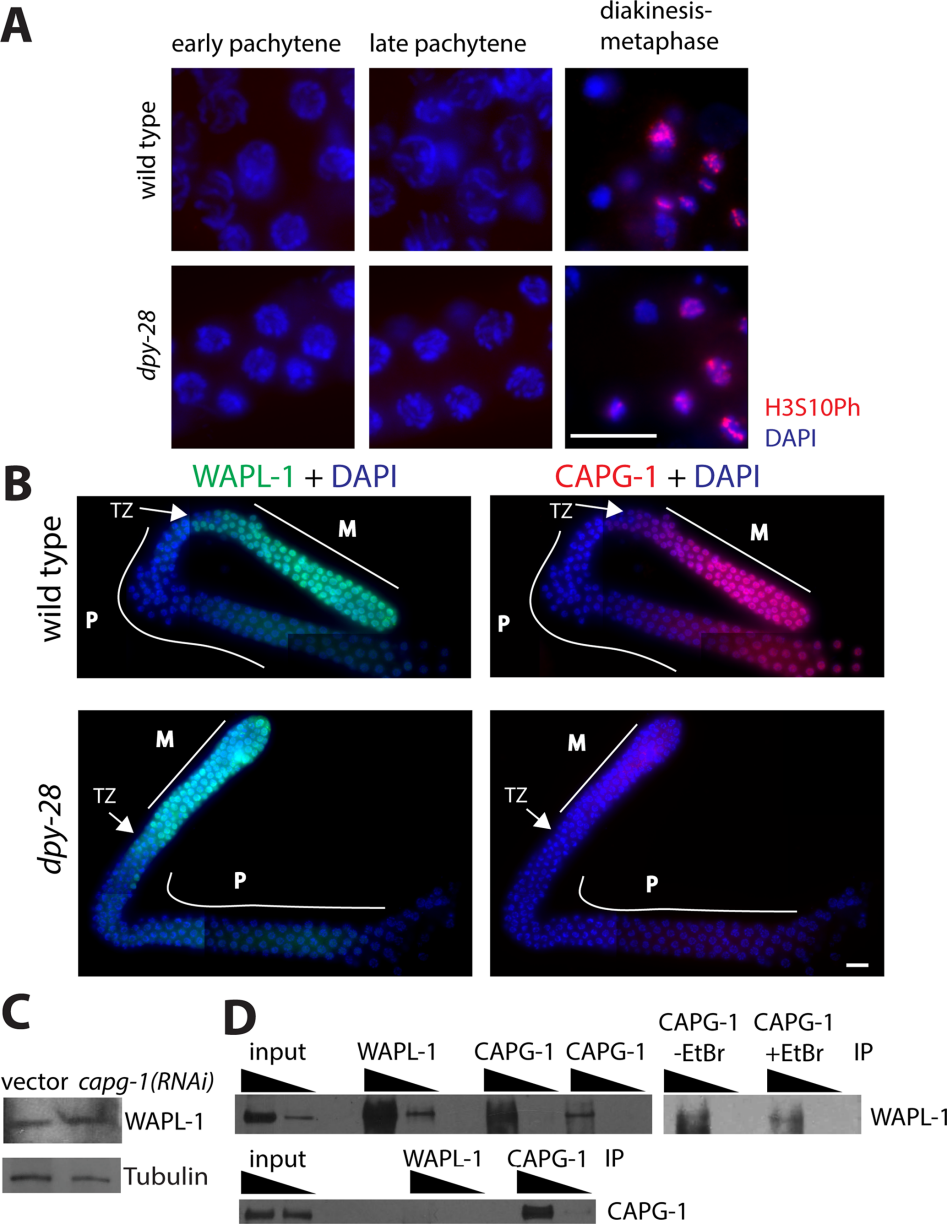
Analysis of WAPL-1 and AIR-2 activity in condensin I mutants. **(A)** Immunofluorescence analysis of H3S10Ph (red) in wild type and *dpy-28(tm3535)* males. DNA is stained with DAPI (blue). H3S10Ph is clearly detectable in diakinesis through metaphase, but levels in pachytene are undetectable both in wild type and in mutants. Scale bar, 10 μm. **(B) I**mmunofluorescence images using antibodies specific to WAPL-1 (green) and condensin I subunit CAPG-1 (red) in wild type and *dpy-28(tm3535)* m-z- male gonads. DNA is stained with DAPI (blue). Scale bar, 10 μm. In wild type males, WAPL-1 and CAPG-1 levels are high in the mitotic tip (M), but are reduced in the transition zone (TZ) and stay low through pachytene (P). In mutants, CAPG-1 is undetectable, but WAPL-1 levels and localization patterns appear unaffected. **(C)** Western blot analysis of control vector and *capg-1* RNAi treated worms expressing a GFP::WAPL-1 transgene. GFP::WAPL-1 protein levels do not change after RNAi. **(D)** WAPL-1 and CAPG-1 interactions analyzed by immunoprecipitation (IP). Top left: IP was performed with anti-WAPL-1, rabbit anti-CAPG1, and goat anti-CAPG-1, and the blot was probed with anti-WAPL-1. Top right: IP was performed with rabbit anti-CAPG-1 with or without added EtBr, and the blot was probed with anti-WAPL-1. Bottom: IP was performed with anti-WAPL-1 and goat anti-CAPG-1, and the blot was probed with rabbit anti-CAPG-1. WAPL-1 was detected in WAPL-1 IPs and in two independent CAPG-1 IPs using different antibodies. Addition of EtBr did not disrupt this interaction. However, CAPG-1 was not pulled down to detectable levels in WAPL-1 IPs.

During meiotic prophase in *C*. *elegans*, AIR-2 promotes cohesin removal [36,37], but this process is normally antagonized by LAB-1 [31]. To determine whether increased AIR-2 activity accounts for the decrease in cohesin levels in condensin I mutants, we tested whether Histone H3S10Ph, a histone mark deposited by active AIR-2, appears prematurely in *dpy-28* males. Accumulation of this mark in early pachytene was previously seen in LAB-1 depleted germlines, correlating with a decrease in cohesin levels [31]. By contrast, we did not observe an increase in H3S10Ph staining in pachytene nuclei of *dpy-28* mutants, even though we were able to detect this mark later in meiosis (Fig 8A). Therefore, we do not have evidence to support the hypothesis of increased AIR-2 activity in condensin I mutants.

Next we tested the role of WAPL-1. Wapl is a cohesin interacting protein implicated in cohesin removal during prophase of mitosis and meiosis in a variety of organisms [17,21,28–30], including *C*. *elegans* [27]. RNAi depletion of *wapl-1* in wild-type males led to a significant increase in COH-3/4 fluorescence compared to empty vector control RNAi, indicating that WAPL liberates COH-3/4*cohesin from chromosomes during male meiosis. The effect of WAPL-1 depletion on REC-8 levels in wild-type males was less significant, consistent with previous observations in hermaphrodites [27] (Fig 1B and 1C). Depleting WAPL-1 by RNAi in *dpy-28* mutant males restored COH-3/4 and REC-8 fluorescence intensity levels to that seen in wild type worms treated with *wapl-1* RNAi (Fig 1B and 1C). These results indicate that WAPL-1 is able to remove both REC-8*cohesin and COH-3/4*cohesin from chromosomes, and that condensin I protects both complexes from WAPL-1 mediated removal.

WAPL-1 and condensin subunit CAPG-1 have similar localization patterns in the germline. Both proteins have diffuse staining patterns in mitotic and meiotic nuclei, with a decrease in signal intensity at the transition zone followed by an increase in late pachytene [27,41,47] (Fig 8B). The similarities in staining patterns are consistent with a direct functional link between the proteins. To test whether condensin I influences WAPL-1 expression, we analyzed GFP::WAPL-1 protein levels in *capg-1*(*RNAi*) worms. GFP::WAPL-1 is expressed from a single copy transgene driven by the *wapl-1* promoter. Expression of this transgene partially rescues *wapl-1* mutant phenotypes [27]. Overall GFP::WAPL-1 protein levels did not change in CAPG-1 depleted worms (Fig 8C). To analyze if WAPL-1 localization is affected by condensin I, we used WAPL-1 specific antibodies to stain the germline of wild type and *dpy-28* mutants, and found that WAPL-1 localization pattern was not altered in mutants (Fig 8B). These results suggest that condensin I may influence WAPL-1 without affecting its expression or localization.

Given the antagonistic interaction between condensin I and WAPL-1 and their similar distributions in the worm gonad, we tested whether condensin I and WAPL-1 physically interact. We performed reciprocal immunoprecipitation experiments using protein extracts prepared from adult males to avoid interactions of condensin I subunits in the context of the dosage compensation complex. Two independently generated CAPG-1 antibodies pulled down WAPL, however WAPL-1-specific antibodies were unable to pull down CAPG-1 (Fig 8D). These results suggest that the interaction between condensin I and WAPL-1 may be weak, or that the interaction between the proteins masks the epitope recognized by the antibody. Addition of Ethidium Bromide (EtBr) to the reaction did not affect the pulldown, suggesting that the interaction may not be DNA-mediated (Fig 8D).

We then assessed whether depletion of *wapl-1* can rescue other defects in *dpy-28* mutants. First we analyzed chromosomal linkages (Fig 2A and 2B). We found that significantly fewer nuclei had two or more 5S rDNA foci in late pachytene of *wapl-1* RNAi treated *dpy-28* mutant germlines compared to empty vector-treated controls, and homolog pairing was restored to nearly wild type levels (Fig 2A and 2B). *wapl-1(RNAi)* also restored chromosomal levels of the SC central element protein SYP-1 (Fig 3D and 3E). These observations suggest that homolog pairing and SC assembly require the condensin I-dependent protection of meiotic cohesin complexes from WAPL-1-mediated removal.

## Discussion

In this study, we identified a previously unknown role for condensin I in protecting chromosomally-bound meiotic cohesin complexes from removal by WAPL-1. In metazoans, meiotic sister chromatid cohesion is mediated by multiple cohesin complexes that differ in their kleisin subunit. Loading and removal of these cohesin complexes is highly dynamic. Sequential cohesin removal by separase-dependent cleavage in anaphase I (to separate homologs) and anaphase II (to separate sister chromatids) has been known for some time. Recently, it has become clear that a substantial portion of cohesin is removed during prophase I, prior to the activation of separase [5,6,10,27,28]. Our results indicate that condensin I regulates the separase-independent prophase removal of cohesin by WAPL-1.

Compaction of mitotic and meiotic chromosomes in all eukaryotes depends on condensin, with most eukaryotes (with the exception of fungi) utilizing two condensins, I and II [65]. In most organisms with two condensins, condensin I plays a role that is equal to or more prominent than that of condensin II. *C*. *elegans* is an exception. The fact that condensin I mutant males survive to adulthood and complete meiosis indicates that both mitosis and meiosis can be completed in the absence of condensin I in this organism. *C*. *elegans* condensin I shares four of five subunits with condensin I^DC^, a gene regulatory complex that is essential in hermaphrodite worms to downregulate gene expression on the two X chromosomes [47,66]. Perhaps as a consequence, condensin II evolved a more prominent role during the processes of cell division and gametogenesis. Alternatively, the unique holocentric nature of *C*. *elegans* chromosomes, with dispersed centromeres along the length of every chromosome, may have imposed different evolutionary pressures on condensin function, as previously suggested [65]. Interestingly, deletion of condensin II, but not condensin I, in the mouse led to a failure of meiosis [67]. It is therefore possible that the prominence of condensin II in meiosis is a conserved feature among metazoans. Our study revealed that although condensin II mutations have more severe phenotypes, condensin I does play an important role in meiosis by regulating cohesin and other aspects of meiotic chromosome structure.

### Condensin I affects meiotic chromosomal axis formation

SC formation is dependent on meiotic cohesin. Cohesin is required for the assembly of the AE (composed of HIM-3, HTP-1/2 and HTP-3), although REC-8*cohesins and COH-3/4*cohesins act partially redundantly in this pathway [3]. The AE is in turn required for the loading of central region proteins SYP-1, -2, -3, and -4 [52,68–71]. Given this hierarchy of assembly, it is interesting that in condensin I mutants (our study) and LAB-1 depleted germlines [31], AE proteins are less affected than SYP-1. Recent superresolution microscopy studies revealed that the AE proteins bridge the region between cohesin and SYP-1 [62]. However, in *rec-8* mutants, the positions of AE proteins shift [62]. In condensin I mutants, cohesin levels are reduced, and it is possible that this reduction leads to a shift in the position of AE proteins, which in turn affects SYP-1 loading. In this model, limited quantities of cohesins are sufficient for AE assembly, but these AEs are not fully functional and cannot support wild type levels of SYP-1 loading. It is also possible that condensin I deficiency affects chromosome structure in some way which leads to not just a decrease in loading, but also a shift in the position of chromosomal cohesins. These cohesin complexes are sufficient to recruit AE proteins, but this altered structure cannot support SYP-1 loading. Higher resolution studies will be needed to distinguish between these possibilities.

### Cohesin regulation during meiotic prophase

Some aspects of the meiotic prophase I cohesin removal mechanism resemble the mitotic "prophase pathway". During mitosis, a non-proteolytic, separase-independent process removes the bulk of cohesin from chromosome arms during prophase. This prophase removal is followed by separase-mediated cleavage of the remaining cohesin at the metaphase to anaphase to transition. The prophase pathway requires the activities of Plk1, Aurora B and Wapl (reviewed in [1]), proteins also implicated in cohesin regulation in meiosis. In *C*. *elegans*, cohesin removal in prophase of meiosis takes place in several waves.

In early prophase I, *C*. *elegans* WAPL-1 appears to antagonize the chromosomal association of meiotic cohesins [27]. REC-8 is expressed prior to entry into meiosis, and REC-8*cohesin forms linear structures called AEs in transition zone nuclei. These nuclei are in leptotene and zygotene, the earliest stages of meiotic prophase. COH-3/4 is undetectable in premeiotic nuclei, but COH-3/4*cohesin appears on chromosomal axes in transition zone nuclei at the same time as REC-8 [10]. In *wapl-1* mutants, these cohesin containing axial structures appear earlier, suggesting that WAPL-1 antagonizes the loading or maintenance of cohesin on meiotic chromosomes [27]. Our data indicates that condensin I counteracts cohesin removal by WAPL-1 at meiotic entry. High levels of COH-3/4 staining were never observed in condensin I mutants, suggesting that the affected step may be loading. Alternatively, cohesins may load onto chromosomes normally but are rapidly removed by WAPL-1, similar to the mechanism used by WAPL-1 in G1.

Interestingly, WAPL-1 and condensin I staining intensity decreases around the same time (in the transition zone) [27,47] (Fig 8) when meiotic cohesin localization defects first appear (S1 Fig). One interpretation is that condensin I and WAPL-1, directly or indirectly, influence the loading of cohesins onto chromosomes in the transition zone, but are not involved in maintenance of cohesins on chromosomes in pachytene. There is an even greater delay in the appearance of pairing defects. While cohesin localization is affected as early as the transition zone, pairing defects become most prominent in mid to late pachytene. This delay may reflect an indirect effect, or it may suggest that in the absence of condensin I the limited quantities of cohesin present on chromosome are sufficient to establish pairing, but are not sufficient for long-term maintenance.

Consistent with condensin I regulating the chromosomal binding of cohesin rather than the overall abundance of cohesin within the nucleus, the earliest decrease in REC-8 staining was detected at entry into meiosis. REC-8 levels were unaffected in premeiotic nuclei, in which REC-8 staining appears nucleoplasmic rather than enriched on chromosomes (S1 Fig). This phenotype is similar to, albeit significantly weaker than, the phenotype of *htp-3* mutants, in which nucleoplasmic REC-8 staining appears normal in premeiotic nuclei, but REC-8 is undetectable in the transition zone and beyond [3]. This similarity suggests that condensin I and HTP-3 may affect the same step in REC-8 loading and/or maintenance immediately upon entry into meiosis.

It is noteworthy that in wild type worms, WAPL-1 depletion mostly affects COH-3/4*cohesins [27] (Fig 1C), but in condensin I mutants, REC-8 and COH-3/4 are affected equally (Fig 1). Thus, WAPL-1 is able to antagonize the chromosomal association of both cohesins, but condensin I counteracts the activity of WAPL-1 toward REC-8 to a greater degree than toward COH-3/4. The mechanisms that load REC-8 and COH-3/4*cohesins onto chromosomes differ. Loading of REC-8*cohesin requires HTP-3 and TIM-1 [3,10]. COH-3/4*cohesin also promotes REC-8 binding, as REC-8 levels are reduced in *coh-4 coh-3* mutants and the signal appears as puncta rather than long threads (Fig 5) [10]. By contrast, COH-3/4 binding is independent of HTP-3 and TIM-1, and its chromosomal loading is not reduced by mutations in *rec-8* [10]. Despite these differences, condensin I influenced the chromosomal association of both types of cohesin. Thus, the antagonistic relationship between condensin I and WAPL-1 determines the levels of REC-8 and COH-3/4 cohesin along the length of meiotic chromosomes throughout early prophase I (leptotene through pachytene).

An additional phase of separase-independent removal of cohesin from chromosomes occurs later in prophase I: in late pachytene and diplotene, WAPL-1 again antagonizes the chromosomal association of cohesin, leading to the accumulation of a nucleoplasmic cohesin pool [27]. Finally, chromosome-associated SMC-1 levels are further reduced in diakinesis between the -2 and the -1 oocyte (the two eldest oocytes); however, this process appears to be independent of WAPL-1 [27]. During late prophase, COH-3/4*cohesin is removed from the long arm and becomes limited to the short arm of bivalents [10]. REC-8 initially localizes to both the long and short arms, but becomes progressively enriched on the long arm, although differences have been noted in previous studies, possibly due to differences in antibodies [10,36,37,72–74]. The process of cohesin removal at this stage is separase independent, and REC-8 removal from the short arm, but not COH-3 removal from the long arm, requires AIR-2 [10]. Whether WAPL-1 influences establishment of reciprocal COH-3/4 and REC-8 domains has not yet been tested. While WAPL-1 plays a role in cohesin regulation both during early and late prophase I, condensin I's role appears to limited to early prophase.

During mitosis, Shugoshin protects cohesin by counteracting the Wapl-mediated prophase pathway [75]. During meiosis I in many organisms, Shugoshin protects centromeric cohesion from separase-mediated degradation [76]. How the activity of a meiotic prophase pathway might be regulated is not known. LAB-1, which forms a complex with HORMA domain proteins HIM-3, HTP-1/2 and HTP-3, was identified as one factor that protects cohesin in early meiosis [31]. We propose that condensin I also promotes cohesin loading and/or maintenance at this stage by antagonizing the activity of WAPL-1. Later in meiosis, LAB-1 [73] and HTP-1/2 [77] and HTP-3 [3], protect REC-8 along the long arm (analogous to centromeric protection in other organisms) to maintain sister chromatid cohesion until anaphase II. Condensin I does not appear to play a role at this stage.

### How does condensin I protect cohesin?

Protection of cohesin by condensin I appears to occur by a different mechanism than that employed by LAB-1. LAB-1 interacts with the PP1 phosphatase homologs GSP-1 and GSP-2 to antagonize the Aurora B kinase AIR-2 [31]. This mechanism is analogous to that employed by Shugoshin, which recruits the PP2A phosphatase to dephosphorylate and thereby protect cohesin from removal [78,79]. We did not observe a change in AIR-2 activity in condensin I mutants (Fig 8), so the mechanism by which condensin I protects meiotic cohesin is likely distinct from that of LAB-1.

Another way Shugoshin protects cohesin from Wapl-dependent removal is by steric hindrance. Analysis of the crystal structure of a cohesin subcomplex revealed that Shugoshin and Wapl compete for the same binding site on cohesin [80], allowing Shugoshin to directly antagonize Wapl by preventing it from making contact with cohesin. We detected physical interactions between WAPL-1 and condensin I (Fig 8), and this interaction may contribute to the ability of condensin I to influence the activity of WAPL-1. A model analogous to physical shielding by Shugoshin would also require that condensin I and cohesin associate with each other (perhaps in a DNA-dependent manner). However, previous biochemical analyses of condensin I interacting proteins did not identify cohesin subunits [41,47], making this possibility less likely. It is however possible that condensin I and cohesin bind to the same site within WAPL-1, such that when WAPL-1 is condensin I-bound, it cannot interact with cohesin. In this model, more WAPL-1 is available to bind and remove cohesin in condensin I mutants than in wild-type animals.

A final possibility is that condensin I influences some aspect of chromosome structure in a manner that promotes the stability of cohesin-DNA interactions. The absence of condensin I would then make cohesin more vulnerable to removal by WAPL-1. Defects in condensin function during *C*. *elegans* meiosis affect chromosome length [47] and level of condensation [81], and these changes may influence the stability of cohesin on DNA. It should be noted that WAPL-1 also influences chromosome axis length in a manner opposite condensin. In *wapl-1* mutants, chromosomes axes are shorter and chromosomes are thicker [27], while in condensin I-depleted animals, chromosomes are longer [47] and less condensed [81]. Therefore, the antagonism between WAPL-1 and condensin I is manifested through opposite effects on both chromosome structure and cohesin regulation. Whether chromosomal association of cohesin influences chromosome structure or chromosome structure influences cohesin binding, or whether these two readouts are independent of each other, remains to be determined.

## Materials and methods

### Strains

*C*. *elegans* strains were cultured at 20°C under standard conditions and maintained on NG agar plates with *E*. *coli* (OP50) as a food source, as described previously [82]. The wild-type strain used in these studies was N2 Bristol, except in experiments using males, in which case wild type control was CB1489 *him-8(e1489) IV*. The *him-8* mutation results in X chromosome nondisjunction without affecting segregation of autosomes, leading to 38% XO male progeny [54]. The following mutant strains were used for this study: EKM-40 *+/hT2 I; dpy-28(tm3535)/hT2 [qIs48] III*, *TY5120 +/nT1 IV; coh-4(tm1857) coh-3(gk112) V/nT1 [qIs51] V*, EKM-92 *rec-8(ok978)/nT1 IV*, *+/nT1 V [qls51] (IV;V)*, TY4986 *htp-3(y428) ccIs4251 I/hT2 [bli-4(e937) let-*?*(q782) qIs48]* (I,III), TY5121 *rec-8(ok978)/nT1 IV; coh-4(tm1857) coh-3(gk112) V/nT1 [qIs51] V*, RB798 *rrf-1(ok589)* I, MD701 *bcIs39 V [lim-7p*::*ced-1*::*GFP + lin-15(+)]*. The GFP::WAPL-1 expressing strain (genotype *fqIs [unc-119(+)*, *wapl-1p*::*GFP*::*wapl-1 II; wapl-1(tm1814) IV* [27]) was a gift from Dr. Enrique Martinez-Perez (Imperial College, London).

The *tm3535* mutation removes 486 bp from exons (end of exon 6 through beginning of exon 8), including 158 bp of exon sequences and introduces a frame shift. The effect on viability is similar to effects of other null and severe loss-of-function alleles of the gene [60]. The EKM-40 *dpy-28(tm3535)/hT2* strain segregates balanced heterozygotes (gfp+) and *dpy-28* homozygotes (gfp-). To isolate *dpy-28* mutant males, non-green (gfp-) homozygous *dpy-28* mutant hermaphrodites from gfp+ heterozygous *dpy-28/+* mothers were collected and allowed to produce the subsequent generation, which consisted of males only. These males have no maternal or zygotic contribution of DPY-28 and presumably lack condensin I function.

### RNAi

RNAi experiments were conducted by feeding worms *E*. *coli* HT115 bacteria transformed with feeding RNAi constructs. The empty parental vector L4440 was used as a control in all RNAi experiments. Clones for *capg-2* and *wapl-1* were obtained from the Ahringer laboratory RNAi feeding library [83]. The template for *capg-1* dsRNA production was amplified from the cDNA clone yk1207f03 using T7 and T3 primers and cloned into L4440. Successful depletions were verified by immunostaining and/or western blotting with the appropriate antibody. Two generation feeding of *capg-1* RNAi bacteria in all genotypes tested was performed as follows: L4-stage hermaphrodites were transferred to feeding RNAi plates (P0 generation), and their progeny (F1 generation) were grown to adulthood and then processed for immunofluorescence. One generation feeding of *capg-2* RNAi bacteria to *rrf-1(ok589)* mutants (S2 Fig) was started at the L1 stage and continued until animals reached adulthood. For *wapl-1* RNAi (Figs 1, 2 and S1), bacteria were fed to adult males for 48 hours prior to tissue dissection and staining.

### Immunofluorescence microscopy

Worms gonads were dissected on poly-L-lysine-coated slides and fixed in 2% PFA, 1X sperm salts (50 mM PIPES pH7.0, 25 mM KCl, 1 mM MgSO_4_, 45 mM NaCl, 2 mM CaCl_2)_, for 5 minutes at room temperature. The slide was covered with a coverslip and incubated on a block of dry ice for 20 minutes. The coverslip was then quickly removed, and slides were washed in PBST (1x PBS with 0.5% Triton X-100) for 10 minutes, 3 times. Primary antibody was diluted in PBST and added to each sample, and slides were incubated overnight at room temperature. Primary antibodies were used at the following dilutions: Anti-COH-3/4 (recognizes both COH-3 and COH-4, [10]) (1:1000), anti-REC-8 (Novus Biologicals cat #29470002, 1:1000), anti-HIM-3 (gift from Dr. Kentaro Nabeshima, University of Michigan, Ann Arbor, MI) (1:2000), anti-HTP-3 [84] (gift from Dr. Abby Dernburg, University of California -Berkeley, Berkeley, CA (1:2000), anti-SCC-1 (Novus Biologicals cat# 29510002; 1:2000), anti-SYP-1 (gift from Dr. Abby Dernburg, University of California -Berkeley, Berkeley, CA) (1:1000), anti-RAD-51 [52] (1:10000), anti-H3S10Ph (6G3; Cell Signaling Technology cat# 9706; 1:500), anti-WAPL-1 (Novus Biologicals, cat# 49300002; 1:2000) and anti-CAPG-1 [41] (1:100). The following day, slides were washed in PBST three times for 10 minutes, then incubated with secondary antibodies at 37°C for 1 hour. Secondary antibodies used in this study were: Cy3 anti-rabbit, Cy3 anti-guinea pig, and FITC anti-rabbit (Jackson Immunochemicals, 1:100). Slides were finally washed three times more with PBST, with the final wash containing DAPI, then mounted with Vectashield (Vector Laboratories).

### Fluorescence in situ hybridization (FISH)

FISH was performed using a 5S rDNA probe and a probe to the left end of chromosome X, derived from DNA purified from a yeast artificial chromosome (YAC) clone, Y02A12. Both probes were prepared, and FISH was performed, as previously described [85].

### Conventional fluorescence microscopy

Images were taken at 0.2 μm increments with an Olympus BX61 motorized Z-drive microscope using a 60x APO oil immersion objective and a Hamamatsu Orca High Resolution Monochrome Cooled CCD (IEEE 1394) camera. Images were processed and analyzed in Slidebook. To standardize image capture, experiments were done in control worms to determine the average (in milliseconds) amount of exposure time for each channel. This exposure time was applied to all samples within the same experiment.

### Quantitative analysis of chromosomal association of cohesin and SYP-1

Fluorescence was quantified on unprocessed images using the Slidebook line intensity and mask tools. For Figs 1 and 3, a square was drawn around the nucleus of interest, and line profiles were generated to show fluorescence intensity. DAPI signal was used as reference for the position of the chromosome. Lines intersected two or more chromosomes. Fluorescence intensity was expressed as the distance from the fluorescence peak to the valley (ΔF) as previously described [27]. For Fig 5B, a “DNA mask” was generated based on DAPI intensity and the “REC-8 mask” was generated based on REC-8 staining intensity. The mask statistics tools was then used to determine the percent of DNA mask covered by the REC-8 mask. Differences in ΔF and coverage values were analyzed by Student’s t-test. Scatter plots depicting ΔF measurements and coverage values were charted using Prism.

### Quantification of FISH foci distances

Distances between FISH foci were measured to determine whether two foci were paired or unpaired. Measurements were carried out using Z-stacks collected at 0.2 increments with 1024x1024 pixel resolution. Distances between peak intensities were determined using the ruler tool in Slidebook. Foci were considered paired when separated by ≤0.75 μm [53,86]. A two-tailed Fisher's exact test or Chi square test was used to evaluate whether differences in the frequency of paired foci in different genotypes were statistically significant.

### Stimulated emission depletion (STED) microscopy

Gonads were dissected as above. Primary antibody was added to each sample overnight at room temperature. Primary antibodies used were rabbit anti-COH-3/4 (diluted 1:1000 in PBST), and guinea pig anti-SYP-1 (1:1000) which was conjugated to goat anti-Guinea Pig IgG (H+L) biotin conjugate antibody (Life Technologies, cat# A18779). The following day, slides were washed 3 times in PBST, for 10 minutes each. Secondary antibody anti-rabbit Oregon Green-488 (ThermoFisher Scientific, cat# O-11038) (1:10000) and V500 Streptavidin (BD Biosciences, cat# 561419) (1:500) were added to slides and allowed to incubate in a humid chamber for two hours at 37°C. Slides again were washed in PBST 3 times for 10 minutes each. Slides were mounted using Glass coverslips (size 1) and Prolong Diamond Mounting medium. All slides were cured for 3–4 days at room temperature followed by one week at -20°C. A Leica SP8 STED scanning confocal imaging system was used for imaging, utilizing a HC PL APO 100x/1.40 OIL STED White objective, a white light continuous wave laser, gated detection, HyD hybrid detectors, a 492 nm depletion laser, and Huygens STED deconvolution. Due to differences in signal intensities between mutant and wild type in the Oregon Green channel, to collect the mutant data, laser intensity was increased from 30% to 36%, and detector gain from 120% to 200%. All other settings, and all experimental manipulations were otherwise identical. These settings were optimized to image existing structures, rather than for the purposes of comparing fluorescence intensities. We attempted to use the settings used for the mutant to acquire wild type images. However, laser intensity could not be fully increased to 36% to avoid overexposing the sensitive HyD detectors. The applied increase in laser intensity and detector gain also led to degradation of resolution in the wild type images. The gap between the two tracks of COH-3/4 staining diminished, making distance measurements hard.

### RNA preparation, reverse-transcription and quantitative PCR

150 adult hermaphrodites were collected in M9 buffer and centrifuged at 13,000 rpm for 1 minute. The supernatant was removed, then worms were stored at -80C. Samples were extracted using TRIzol and cleaned using the RNeasy Mini Kit (Qiagen, cat# 74104), then treated with DNase (Qiagen, RNase-Free DNase Set, cat# 79254). cDNA was synthesized using the VILO master mix system (ThermoFisher cat# 11755250), then stored at -20°C. 20 ng cDNA template was used for real-time quantitative PCR amplification using PowerUp (ThermoFisher cat# A25741) in a 15 μl reaction volume. Reactions were run in MicroAmp Fast Optical 96-Well Reaction Plates (cat#4346907) in a StepOne Plus qPCR (Applied Biosystems) cycler. Sequences for qPCR primers were: GCAAGCAAGCTCATTCAGTGG and CCGTACACCAAATTACACGCAA to detect *coh-3* and *coh-4* transcripts, and AACTCCAGAGAAACGCCGG and GTCGATTACGGCGAGTATCCT to detect *rec-8* transcript levels. Expression levels of each gene were analyzed using the ΔΔ2Ct method, normalized to the *pmp-3* gene, using primers GTTCCCGTGTTCATCACTCAT and ACACCGTCGAGAAGCTGTAGA. Measurements were performed in duplicates with three biological replicates for each condition.

### Immunoprecipitation

Packed adult males were frozen in liquid nitrogen, ground using mortar and pestle, scraped into cold PBS with protease inhibitors, and lysed by sonication as described previously [41]. 3 mg of protein extract was used for each IP. Protein A (for rabbit antibodies) or Protein G (for goat antibodies) Dynabeads (Thermo Fisher, cat# 10001D and 10003D) were incubated with 5 μg of primary antibody for 4 hours at 4°C. Protein lysate was precleared with IgGsorb (The Enzyme Center) for one hour at 4°C. Beads were washed with PBS, then PBST, added to precleared protein lysate and incubated at 4°C overnight. When indicated, EtBr to a final concentration of 50 μg/mL was added prior to the overnight incubation. Beads were then washed, and samples were analyzed by western blotting, as below.

### Western blot

100 adult worms were collected in M9 buffer with protease inhibitors, then frozen for 24 hours. An equal volume of sample buffer was added (0.1 M Tris pH 6.8, 7.5M urea, 2% SDS, 100mM ME, 0.05% bromophenol blue), and the mixture was heated to 65°C for 10 minutes, sonicated for 30-seconds twice, heated to 65°C for 5 minutes, 95°C for 5 minutes, then kept at 37°C until loading onto SDS-PAGE gel. For analysis of IP samples, beads from above were incubated with sample buffer and heated to 65°C for 10 minutes, then 95°C for 5 minutes, then loaded onto gels. Proteins were transferred to nitrocellulose and probed with rabbit anti-CAPG-1 [41] (Figs 1 and 8) or goat anti-CAPG-1 [87] (Fig 8), anti-GFP (Roche, 11 814 460 001) to assess GFP::WAPL-1 levels, anti-WAPL-1 (Novus Biologicals, cat# 49300002), and anti-beta tubulin (Novus Biologicals, NB600-936) for loading control. Secondary antibodies were anti-rabbit-HRP or anti-goat-HRP (Jackson Immunochemicals).

## Supporting information

S1 Fig Condensin I affects cohesin localization at entry into meiosis. **(A)** Immunofluorescence images of COH-3/4 and REC-8 staining in the mitotic tip (MT) and transition zone (TZ) of wild type and *dpy-28* mutant male germlines treated with control vector or *wapl-1* RNAi. COH-3/4 is not detectable in the MT, and first appears on chromosomes in the TZ. Staining intensity is reduced in the TZ in *dpy-28* mutants. REC-8 is nucleoplasmic in the MT and appears as long threads on chromosomes in the TZ. In *dpy-28* mutants, localization patterns are unchanged in the MT, but staining intensity is reduced in TZ. *wapl-1* RNAi restores cohesin staining intensity to near wild type levels. Scale bar, 5 μm **(B)** Metaphase I stage in wild type and *dpy-28* mutant males. REC-8 and COH-3/4 are shown in green. Cohesin staining intensities and localization appear similar in wild type and mutant, with COH-3/4 enriched between paired homologs (arrows). Scale bar, 1 μm.
(TIF)

S2 Fig Cohesin and SC localization in condensin I depletion and condensin II mutants. **(A)** Immunofluorescence images of REC-8 and COH-3/4 staining in early pachytene (EP), and late pachytene (LP) nuclei of *rrf-1* hermaphrodites treated with control vector or *capg-1* RNAi. Chromosomal association of REC-8 and COH3/4 is reduced after CAPG-1 depletion. **(B)** Control experiment showing lack of COH-3/4 staining in *coh-4 coh-3* mutants and lack of REC-8 staining in *rec-8* mutants. **(C)** Images of single bivalents (paired homologs) at diakinesis in *rrf-1* hermaphrodites. COH-3/4 (green) is enriched at the short arm of bivalents (between homologs, arrows). and REC-8 (green) is initially visible on both arms, but eventually is more prominent on the long arm (between sisters, arrows). Staining patterns are comparable in control and in *capg-1(RNAi)*. **(D)** Immunofluorescence images of gonads from control and *capg-2* RNAi-treated worms stained with antibodies specific for SYP-1 (red) and COH-3/4 (green) on the left and HTP-3 (red) and REC-8 (green) on the right. DNA is stained with DAPI (blue). Depletion of CAPG-2 did not perturb cohesin or SC localization. Scale bars, 5 μm in A, B, and D, and 1 μm in C.
(TIF)

S3 Fig Double strand DNA break repair and apoptosis in condensin I-depleted germlines. **(A)** Immunofluorescence images of wild type male and *dpy-28(tm3535)* male gonads stained with antibodies specific to double strand DNA break marker RAD-51. Scale bar, 5 μm. **(B)** Quantification of RAD-51 foci in different zones of the male germline. In *dpy-28* mutants, the number of foci increase, particularly in early pachytene (EP) and mid pachytene (MP). By late pachytene, breaks are resolved in both genotypes. Numbers of nuclei analyzed and p values are shown in S1 Table. **(C)** Quantification of apoptotic nuclei in hermaphrodites expressing the apoptosis marker CED-1::GFP, treated with control vector or *capg-1* RNAi. Total numbers of apoptotic bodies per gonad arm are shown. Germline apoptosis increases after *capg-1* RNAi. *** indicates statistical significance (p<0.001) by two-tailed unpaired t-test.
(TIF)

S4 Fig SYP-1 aggregates are observed in *htp-3* mutants. Immunofluorescence images in wild type and *htp-3* mutant hermaphrodite gonads stained with SYP-1 antibodies. SYP-1 forms long tracks along chromosomes in wild type worms, but it is present in aggregates in *htp-3* mutants.
(TIF)

S1 Table Numerical and statistical data. Numerical data underlying graphs and summary statistics.
(XLSX)
