# Supplementary figures and images for: Condensin I protects meiotic cohesin from WAPL-1 mediated removal
Source: PLoS Genet. 2018 May 16;14(5):e1007382. doi: 10.1371/journal.pgen.1007382 (PMC5973623; doi:10.1371/journal.pgen.1007382)

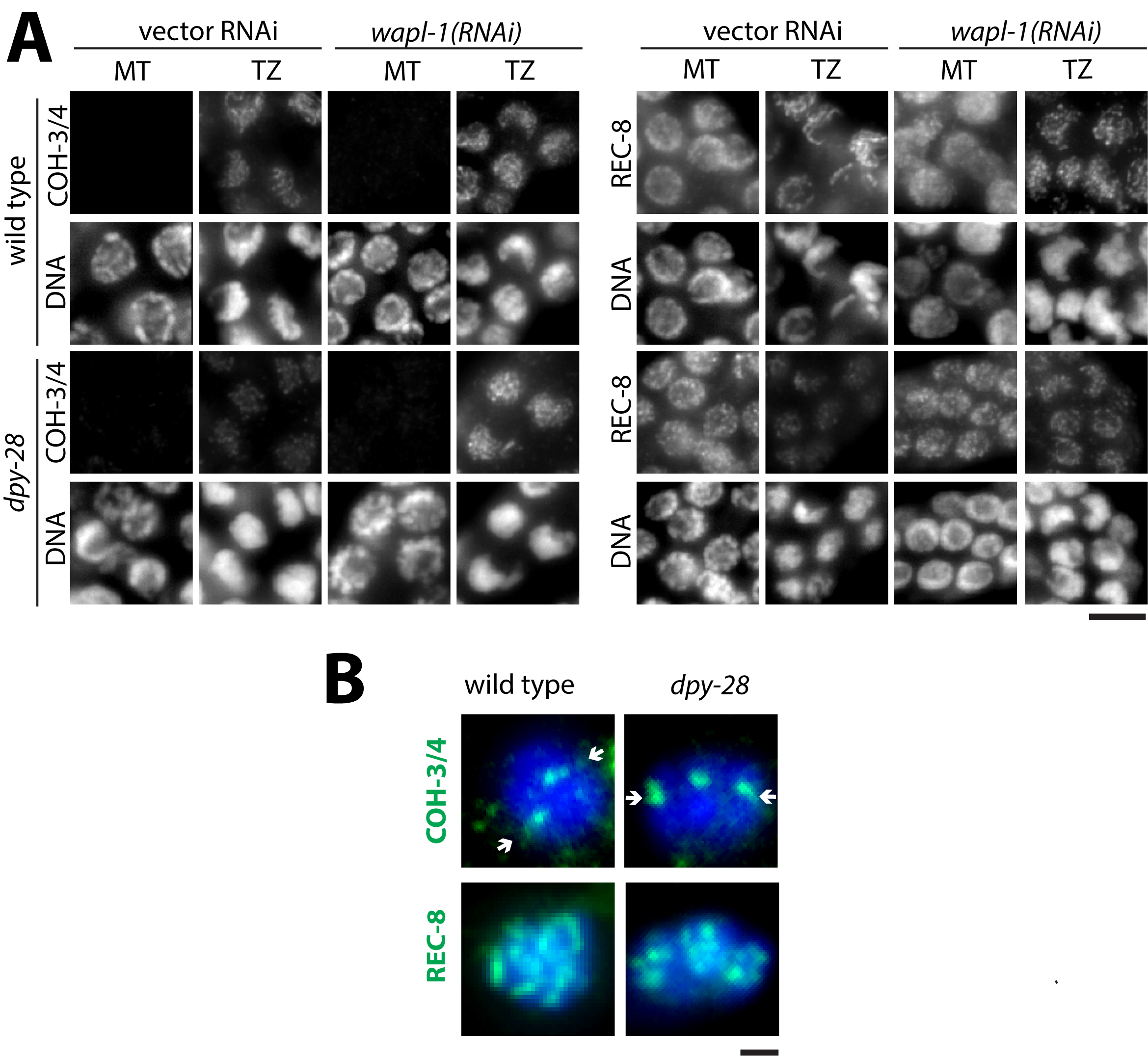

Supplement: S1 Fig — (A) Immunofluorescence images of COH-3/4 and REC-8 staining in the mitotic tip (MT) and transition zone (TZ) of wild type and dpy-28 mutant male germlines treated with control vector or wapl-1 RNAi. COH-3/4 is not detectable in the MT, and first appears on chromosomes in the TZ. Staining intensity is reduced in the TZ in dpy-28 mutants. REC-8 is nucleoplasmic in the MT and appears as long threads on chromosomes in the TZ. In dpy-28 mutants, localization patterns are unchanged in the MT, but staining intensity is reduced in TZ. wapl-1 RNAi restores cohesin staining intensity to near wild type levels. Scale bar, 5 μm (B) Metaphase I stage in wild type and dpy-28 mutant males. REC-8 and COH-3/4 are shown in green. Cohesin staining intensities and localization appear similar in wild type and mutant, with COH-3/4 enriched between paired homologs (arrows). Scale bar, 1 μm. (TIF) [file pgen.1007382.s001.tif]

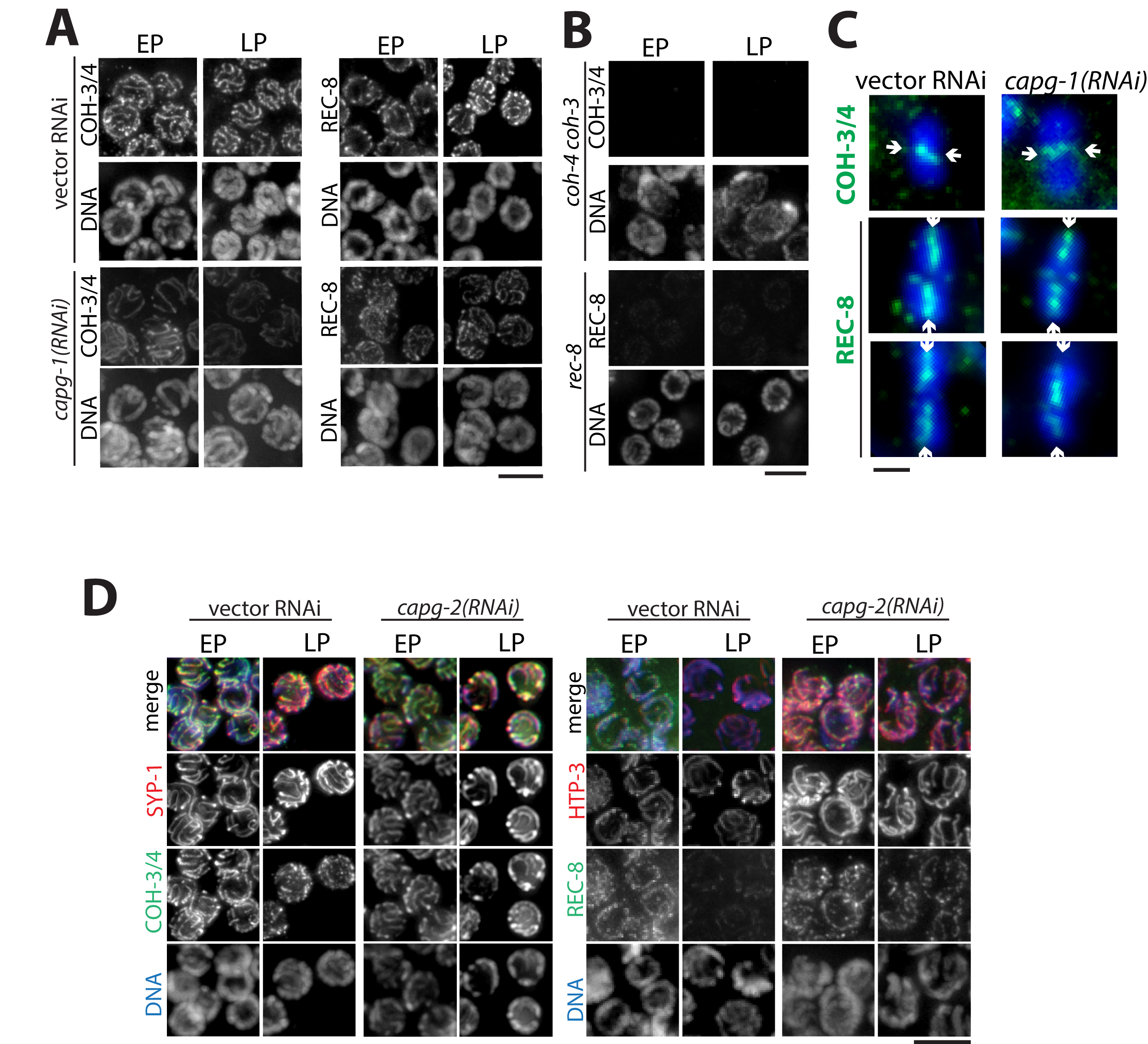

Supplement: S2 Fig — (A) Immunofluorescence images of REC-8 and COH-3/4 staining in early pachytene (EP), and late pachytene (LP) nuclei of rrf-1 hermaphrodites treated with control vector or capg-1 RNAi. Chromosomal association of REC-8 and COH3/4 is reduced after CAPG-1 depletion. (B) Control experiment showing lack of COH-3/4 staining in coh-4 coh-3 mutants and lack of REC-8 staining in rec-8 mutants. (C) Images of single bivalents (paired homologs) at diakinesis in rrf-1 hermaphrodites. COH-3/4 (green) is enriched at the short arm of bivalents (between homologs, arrows). and REC-8 (green) is initially visible on both arms, but eventually is more prominent on the long arm (between sisters, arrows). Staining patterns are comparable in control and in capg-1(RNAi). (D) Immunofluorescence images of gonads from control and capg-2 RNAi-treated worms stained with antibodies specific for SYP-1 (red) and COH-3/4 (green) on the left and HTP-3 (red) and REC-8 (green) on the right. DNA is stained with DAPI (blue). Depletion of CAPG-2 did not perturb cohesin or SC localization. Scale bars, 5 μm in A, B, and D, and 1 μm in C. (TIF) [file pgen.1007382.s002.tif]

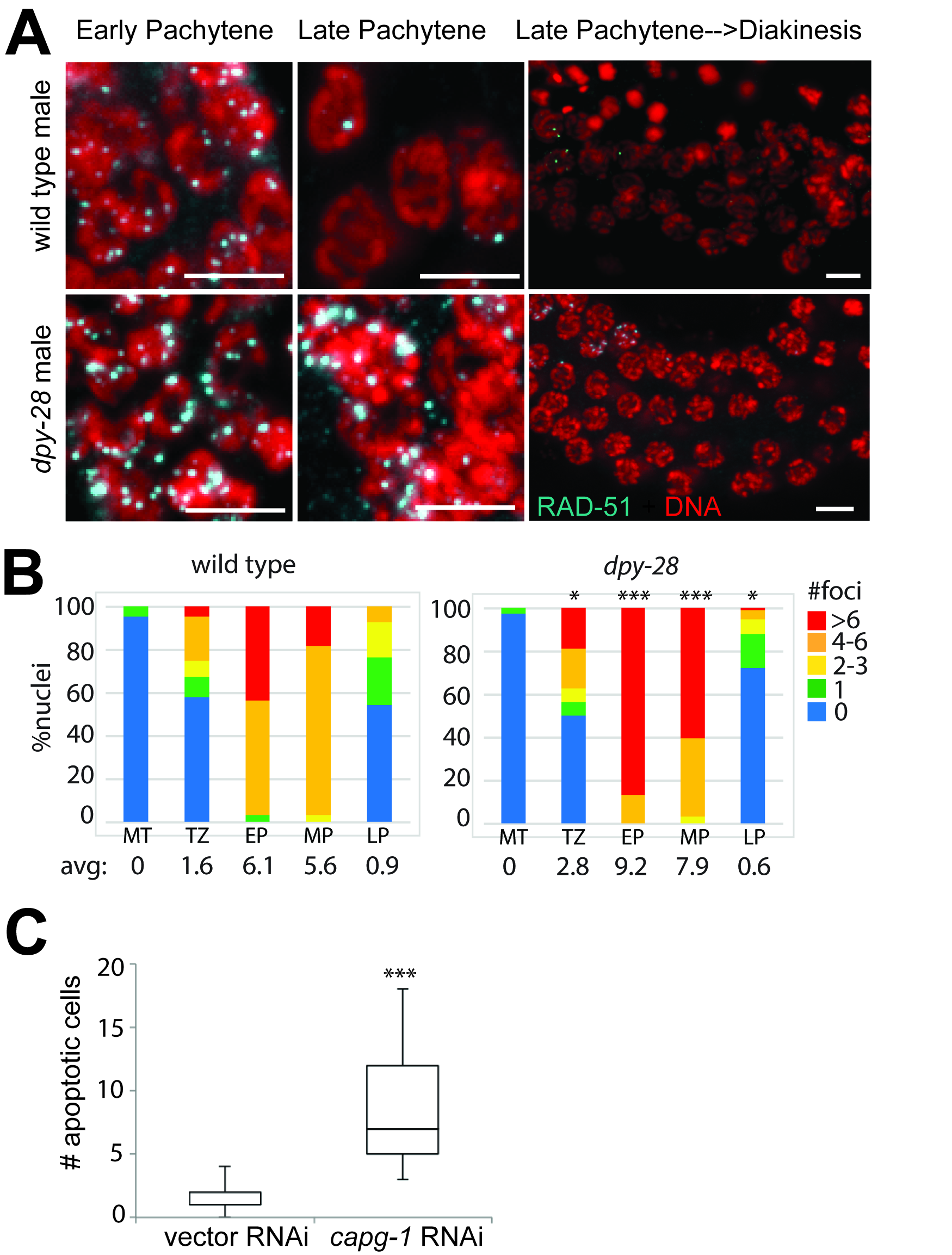

Supplement: S3 Fig — (A) Immunofluorescence images of wild type male and dpy-28(tm3535) male gonads stained with antibodies specific to double strand DNA break marker RAD-51. Scale bar, 5 μm. (B) Quantification of RAD-51 foci in different zones of the male germline. In dpy-28 mutants, the number of foci increase, particularly in early pachytene (EP) and mid pachytene (MP). By late pachytene, breaks are resolved in both genotypes. Numbers of nuclei analyzed and p values are shown in S1 Table. (C) Quantification of apoptotic nuclei in hermaphrodites expressing the apoptosis marker CED-1::GFP, treated with control vector or capg-1 RNAi. Total numbers of apoptotic bodies per gonad arm are shown. Germline apoptosis increases after capg-1 RNAi. *** indicates statistical significance (p<0.001) by two-tailed unpaired t-test. (TIF) [file pgen.1007382.s003.tif]

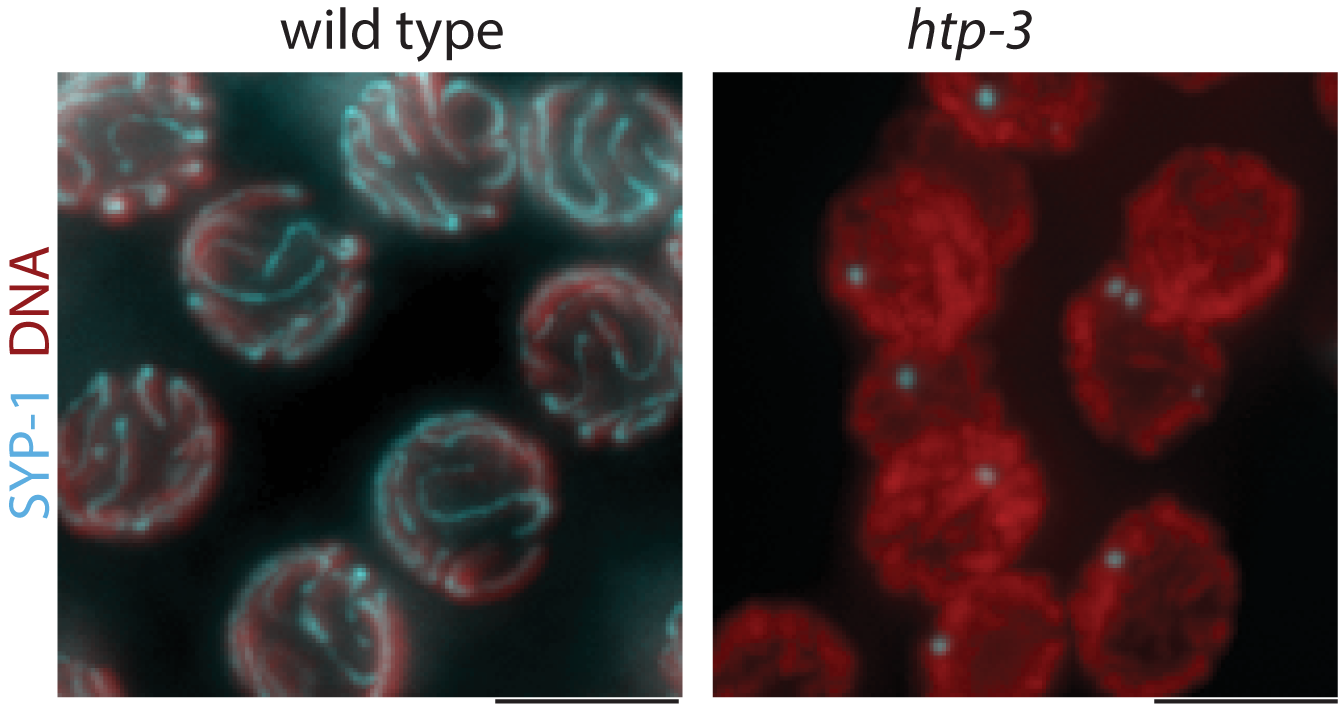

Supplement: S4 Fig — Immunofluorescence images in wild type and htp-3 mutant hermaphrodite gonads stained with SYP-1 antibodies. SYP-1 forms long tracks along chromosomes in wild type worms, but it is present in aggregates in htp-3 mutants. (TIF) [file pgen.1007382.s004.tif]
